# High-Throughput Native Mass Spectrometry Screening in Drug Discovery

**DOI:** 10.3389/fmolb.2022.837901

**Published:** 2022-04-14

**Authors:** Agni F. M. Gavriilidou, Kleitos Sokratous, Hsin-Yung Yen, Luigi De Colibus

**Affiliations:** OMass Therapeutics, Oxford, United Kingdom

**Keywords:** Native Mass Spectrometry, Structural Biology, Drug Discovery, Screening, Affinity

## Abstract

The design of new therapeutic molecules can be significantly informed by studying protein-ligand interactions using biophysical approaches directly after purification of the protein-ligand complex. Well-established techniques utilized in drug discovery include isothermal titration calorimetry, surface plasmon resonance, nuclear magnetic resonance spectroscopy, and structure-based drug discovery which mainly rely on protein crystallography and, more recently, cryo-electron microscopy. Protein-ligand complexes are dynamic, heterogeneous, and challenging systems that are best studied with several complementary techniques. Native mass spectrometry (MS) is a versatile method used to study proteins and their non-covalently driven assemblies in a native-like folded state, providing information on binding thermodynamics and stoichiometry as well as insights on ternary and quaternary protein structure. Here, we discuss the basic principles of native mass spectrometry, the field’s recent progress, how native MS is integrated into a drug discovery pipeline, and its future developments in drug discovery.

## Introduction

The investigation of non-covalent interactions between a biological macromolecule and small molecule, which are driven by a myriad of forces including hydrogen bonds, Van der Waals forces, electrostatic and hydrophobic interactions, play a crucial role in the development of drug candidates. A variety of analytical methods are utilized to identify and quantify protein-ligand interactions, including isothermal titration calorimetry (ITC) (de Azevedo and Dias, 2008), surface plasmon resonance (SPR) spectroscopy (Cooper, 2002), nuclear magnetic resonance (NMR) spectroscopy (Meyer and Peters, 2003), frontal affinity chromatography combined with mass spectrometry (Slonusakiewicz et al., 2005), ThermoFluor and enzyme-linked immunosorbent assays (Benesch and Ruotolo, 2011; Aebersold and Mann, 2016).

The mass spectrometry (MS) analytical toolbox contains numerous biophysical techniques (Benesch and Ruotolo, 2011), but only a few are used in a high-throughput manner for drug discovery. One example is the analysis of biomolecules and their assemblies using MS-based proteomics approaches, which can provide means for proteome-wide quantitation of proteins, monitor their levels, and characterize protein-protein interactions and post-translational modifications (Aebersold and Mann, 2016). Another technique that has become a valuable complement to X-ray crystallography in determining protein structure, dynamics and identification of small molecule binding sites is hydrogen/deuterium exchange (HDX) coupled with mass spectrometry (Smith et al., 1997; Chalmers et al., 2011; Konermann et al., 2011; Engen and Wales, 2015; Masson et al., 2019). Both techniques are widely used for the study of proteins and biomolecules in final denaturing conditions. Native MS, the core focus of this review, represents an addition to the analytical toolbox of mass spectrometry and has, over the past decade, experienced immense growth. It is used for studying intact proteins and their complexes, including interactions with small-molecule drugs, in a native-like folded state. Unlike the analytical tools outlined above, native MS enables the investigation of non-covalent interactions, without the need for labelling or crosslinking, using just picomoles of material (Laganowsky et al., 2013), while at the same time offering high-resolution (Rose et al., 2012; Gault et al., 2016) and a speed of analysis in the timescale of milliseconds (Breuker and McLafferty, 2008).

Native MS exploits the gentle nature of electrospray ionization (ESI) (Fenn et al., 1989) to transfer proteins and their non-covalent complexes from volatile buffered aqueous solutions into the gas-phase. Under controlled operating conditions within the mass spectrometer the biomolecules can retain a near native-like structure and quaternary non-covalent interactions can be preserved (Khristenko et al., 2019). Shortly after the development of ESI-MS in the late 1980s (Fenn et al., 1989), successful applications demonstrating its potential use as a tool for probing non-covalent protein complexes were reported. Early examples include the detection of the intact heme-myoglobin complex in 1991 (Katta and Chait, 1991) and the non-covalent complex between the cytoplasmic receptor FKBP with the immunosuppressive agents FK506 and rapamycin (Ganem et al., 1991), in the same year. The initial studies demonstrating the capability of ESI-MS to preserve protein-ligand interactions, were followed by studies utilizing ESI-MS for the quantification of protein-ligand binding affinities. In 1993, Loo and co-workers reported the enthalpy of dissociation (ΔH) of the ribonuclease S-protein—S-peptide complex, and the values of the dissociation constant (K_d_) of the same complex over a range of temperatures (Loo et al., 1993). Furthermore, the ability of ESI-MS to measure non-covalently bound protein assemblies, like the ribosome, a 2 megaDalton (MDa) protein-RNA complex (McKay et al., 2006), the tetradecameric GroEL (Sobott and Robinson, 2006) and the 20S proteasome, a 690 kiloDalton (kDa) 28-mer (Loo et al., 2005) has also been successfully demonstrated during the years. Although these examples for the preservation of non-covalent interactions during ESI (Fenn et al., 1989) were received with great enthusiasm, they raised at the same time the question of whether or not ESI (Fenn et al., 1989) could preserve the native solution structure of biomolecules and their assemblies during transfer into the gas-phase (Wolynes, 1995). Even almost 30 years after these initial demonstrations, there is much debate on the structure of protein ions in gas-phase. Clearly, the new gaseous environment could cause dramatic structural rearrangements; however, the question is how long it takes to transform solution structure to solvent-free structure and whether or not this is within the time frame of the ESI process and MS analysis. Whilst it appears probable that surface side-chain collapse occurs within picoseconds of dehydration, some elements of gross structural rearrangement may require milliseconds or even more (Breuker and McLafferty, 2008). For example, an ion mobility spectrometry (IMS) study showed cytochrome C gas-phase ions’ unfolding in the milliseconds region (Badman et al., 2005). Another IMS study by Wyttenbach and Bowers has investigated the structural stability of ubiquitin during the transition from solution to gas-phase. The authors concluded that during the ESI process, the native state of ubiquitin is preserved. Moreover, ubiquitin can survive for more than 100 milliseconds in a 294 K solvent-free environment (Wyttenbach and Bowers, 2011). Therefore, there may be a window for observing gas-phase ion species, which are relatively similar to their solution structures. Several other studies have also suggested that gas-phase ions generated during the ESI process can retain significant aspects of their solution structures (Ruotolo and Robinson, 2006; Koeniger and Clemmer, 2007; Bernstein et al., 2009; Grabenauer et al., 2010; Breuker et al., 2011).

Over the last 30 years, numerous examples have proven the analytical advantages of native MS (Fenn et al., 1989) in addressing biological questions such as stoichiometry determination (Rostom and Robinson, 1999; Rostom et al., 2000; Geels et al., 2006), oligomeric state formation (Hernández et al., 2006; Zhou et al., 2008), allostery (Dyachenko et al., 2013; Gavriilidou et al., 2018a), application to amyloids (Santambrogio et al., 2011; Bleiholder et al., 2013), antibodies (Thompson et al., 2014; Terral et al., 2016) and membrane proteins (Gupta et al., 2018; Robinson, 2019; Keener et al., 2021). Here, we will discuss the current application of native MS in ligand/drug screening, its latest advances, especially in the field of membrane proteins, which represent the majority of current pharmacological targets and the future option to integrate native MS with other structural biology techniques to shed light on the mechanism of action of a drug candidate *in situ*.

## Instrumentation of Native Mass Spectrometry

ESI (Fenn et al., 1989) is the consequence of the application of electricity to an ion-containing liquid that undergoes a series of subsequent evaporation and droplet shrinking events, which in turn lead to its dispersion into a fine jet. The fundamental principles of ESI have been extensively studied to date (Kebarle and Verkerk, 2009; Konermann et al., 2013). Briefly, the three prevailing models are the charged-residue model (CRM) (Kebarle and Peschke, 2000), the ion evaporation model (IEM) (Iribarne and Thomson, 1976; Thomson and Iribarne, 1979) and the chain ejection model (CEM) (Konermann et al., 2013). Evidence from ESI-MS results obtained on a variety of globular proteins studied under native conditions support that the CRM mechanism is followed in the case of native MS and it is currently the most widely accepted framework for the modelling of ESI (Fernandez De la Mora, 2000; Heck and van den Heuvel, 2004; Nesatyy and Suter, 2004). The CEM applies to unfolded proteins that are particularly hydrophobic and also capable of accommodating excess charges, whereas the IEM mechanism has been suggested to be followed in the case of small molecules such as peptide ions.

**FIGURE 1:**
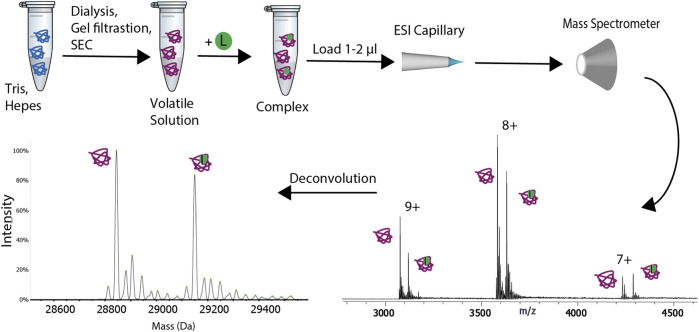
Schematic representation of the experimental procedure for native mass spectrometry. The sample is transferred from its buffer to a volatile solution. Buffer exchange can be performed *via* one of the following methods: gel filtration, size exclusion chromatography (SEC) or dialysis. The sample is then loaded on a ESI or nanoESI capillary and it is transferred to the gas-phase. The ions are subsequently analyzed with the mass spectrometer. A ligand (L), represented with a green circle can be added to the buffer exchanged sample. The deconvolution of the spectra will result in two peaks, one for the apo protein and the other for the complex.

The ionization of proteins and protein assemblies by ESI (Fenn et al., 1989) generates multiple charge states, which are visualized in a mass spectrum by a series of peaks, each of a specific mass-to-charge ratio (*m/z*). The collection of charge states that represent a single protein moiety, is referred as the charge state distribution (CSD) of gas-phase protein ions, and usually resembles one or more Gaussian distributions (Borysik et al., 2004) (Figure 1). The extent of CSD observed in an ESI spectrum depends on the solvent-exposed surface area of the protein, with more compact structures, such as those that can be obtained in a native MS spectrum, acquiring fewer charges (Konermann and Douglas, 1998; Fenselau et al., 2000). Multiple Gaussian distributions for a single species may indicate the presence of more than one solution conformation (Chowdhury et al., 1990; Dobo and Kaltashov, 2001). Overall, solution conditions and experimental parameters have to be tuned carefully, and it has been shown that they affect how molecules are transferred to the gas-phase (Benkestock et al., 2004; Peschke et al., 2004). For example, protein unfolding during the ESI (Fenn et al., 1989) process has been related to increased charge states (Shelimov et al., 1997). The unfolding at higher charge states is attributed to increased intra- and intermolecular Coulomb repulsion within a protein ion. It can be assumed that lower charge states minimize structure unfolding, leading to the formation of more native-like species. Moreover, solution additives or impurities could lead to the formation of adducts with alkali metal cations, such as sodium and potassium, which can destabilize a protein-ligand system during the ESI process by lowering the activation barrier to dissociation (Hopper and Oldham, 2011). However, other studies have suggested that alkali metals can actually stabilize gas-phase protein structures *via* the formation of additional interactions (Wu et al., 1999; Rožman and Gaskell, 2010).

A miniaturized version of ESI (Fenn et al., 1989), known as nano-ESI, was introduced in 1994 by Wilm and Mann (Wilm and Mann, 1994). Its advantages over conventional ESI are higher sensitivity, better resolution, increased tolerance to non-volatile salts (Lössl et al., 2014; Susa et al., 2017) and low sample consumption (Juraschek et al., 1999; Kebarle and Verkerk, 2009). Moreover, nano-ESI (Wilm and Mann, 1994) is also more sensitive and tolerant to buffer contaminants than conventional ESI (Wilm and Mann, 1996), and since the droplets formed by nano-ESI are smaller, low source/desolvation temperatures can be used, enabling better preservation of non-covalent interactions, which may be destabilized as a result of heating (Hernández and Robinson, 2007). The most commonly used volatile buffer is ammonium acetate which has a pH range of 6–8 and evaporates readily during ionization (Hernández and Robinson, 2007; Gavriilidou et al., 2015).

Nano-ESI has also been shown to be applicable to the analysis of membrane proteins. However, when working with membrane proteins, the sample must be supplemented with detergent or transferred to the gas-phase *via* lipid-based vehicles such as nanodiscs, amphipols or bicelles (Hopper et al., 2013; Laganowsky et al., 2013). For a more in depth focus on new analytical advances and application of native MS on membrane proteins we direct the readers to a recent review by Keener et al. (2021).

Sample ionization followed by separation of ions according to their *m/z* ratio and finally, ion detection are the three key steps that are followed in every MS experiment.

Several types of MS instruments are currently available, offering analytical sensitivity, specificity and speed in the analysis of mainly small molecules, such as drugs or peptides, as well as analysis of large therapeutic molecules (Rathore et al., 2018) or even larger macromolecules, such as viral capsids, with molecular weights in the MDa mass range (Snijder et al., 2013).

As mentioned above, native MS analysis results in a relatively narrow charge state distribution of gas-phase ions with fewer charges than the same system would attain under denaturing conditions (Konermann and Douglas, 1998). Hence, native MS analysis of proteins and protein assemblies requires mass analyzers that can operate at a higher *m/z* range and at the same time be optimized for the efficient transmission of large molecular ions carrying a relatively low number of charges. The first mass analyzer adapted for native MS experiments was the time of flight (ToF) (Mirgorodskaya et al., 1994) mass analyzer, which has a theoretically unlimited *m/z* range. Modified ToF-based instruments dominated the field of native MS for over 2 decades. However, more recently, Fourier transform ion cyclotron resonance (FT-ICR) and Orbitrap mass analyzers, have also been adapted for native MS (Zhang et al., 2011; Rose et al., 2012).

Many modern MS-instruments used for native MS combine these mass analyzers with either a quadrupole or an ion trap mass analyzer, in a single configuration. One of the most common hybrid instruments extensively used in studies of biomolecular assemblies is the Quadrupole Time–of–flight (QTof) (Morris et al., 1996), which consists of a quadrupole filter, a collision cell and a ToF analyzer. Recently, an Orbitrap mass analyzer with ultrahigh mass range (UHMR) featured with a quadrupole analyzer has been developed for native MS (Van De Waterbeemd et al., 2017; Fort et al., 2018). An Orbitrap-based trihybrid instrument featuring both a quadrupole and a linear ion trap mass analyzer has also been added to MS instruments capable of native MS studies (Gault et al., 2020).

## Determination of Protein-Drug Interactions

Native MS is used to study a wide diversity of biological samples that differ in mass, polydispersity, symmetry and dynamic flexibility (Kaur et al., 2019). This brings tremendous analytical advantages in interrogating protein-drug interactions. Different oligomeric states can be investigated simultaneously with no need for labelling or crosslinking. Specific information is obtained for each individual species present, without data being averaged over different species. Native MS can distinguish by mass and thus reveal the entire distribution of ligand-bound states. Therefore, the dynamics of quaternary structure can be studied in real time (Yee et al., 2019). Gavriilidou et al. (2018a) demonstrated this advantage of native MS in the study of the dimer−tetramer equilibrium of M2 pyruvate kinase (PKM2), a regulatory enzyme that is often inactive in the glycolytic pathway in tumor cells. An allosteric activator, fructose-1,6- bisphosphate (FBP), was found to shift the dimer−tetramer equilibrium toward the active tetramer, with the 4:4 stoichiometry of FBP binding to the tetramer only. Other than revealing multimeric concomitant binding, native MS helps distinguish allosteric mechanisms as has been shown by the Sharon group (Dyachenko et al., 2013) for the ligation pathway of ATP to GroEL. Moreover, their approach was able to discriminate between the Monod–Wyman–Changeux (Jacques et al., 1965) and the Koshland–Némethy–Filmer (KNF) allosteric models (Koshland et al., 1965). Native MS showed its strength as well in analyzing complexes of proteins with covalently bound molecules (Lu et al., 2021). For example, Douangamath et al. conducted a combined mass spectrometry and high throughput crystallographic fragment screen against SARS-CoV-2 main protease (M^pro^), using over 1,250 fragments from a compound library that yielded 48 high-value covalent fragments (Douangamath et al., 2020).

**FIGURE 2:**
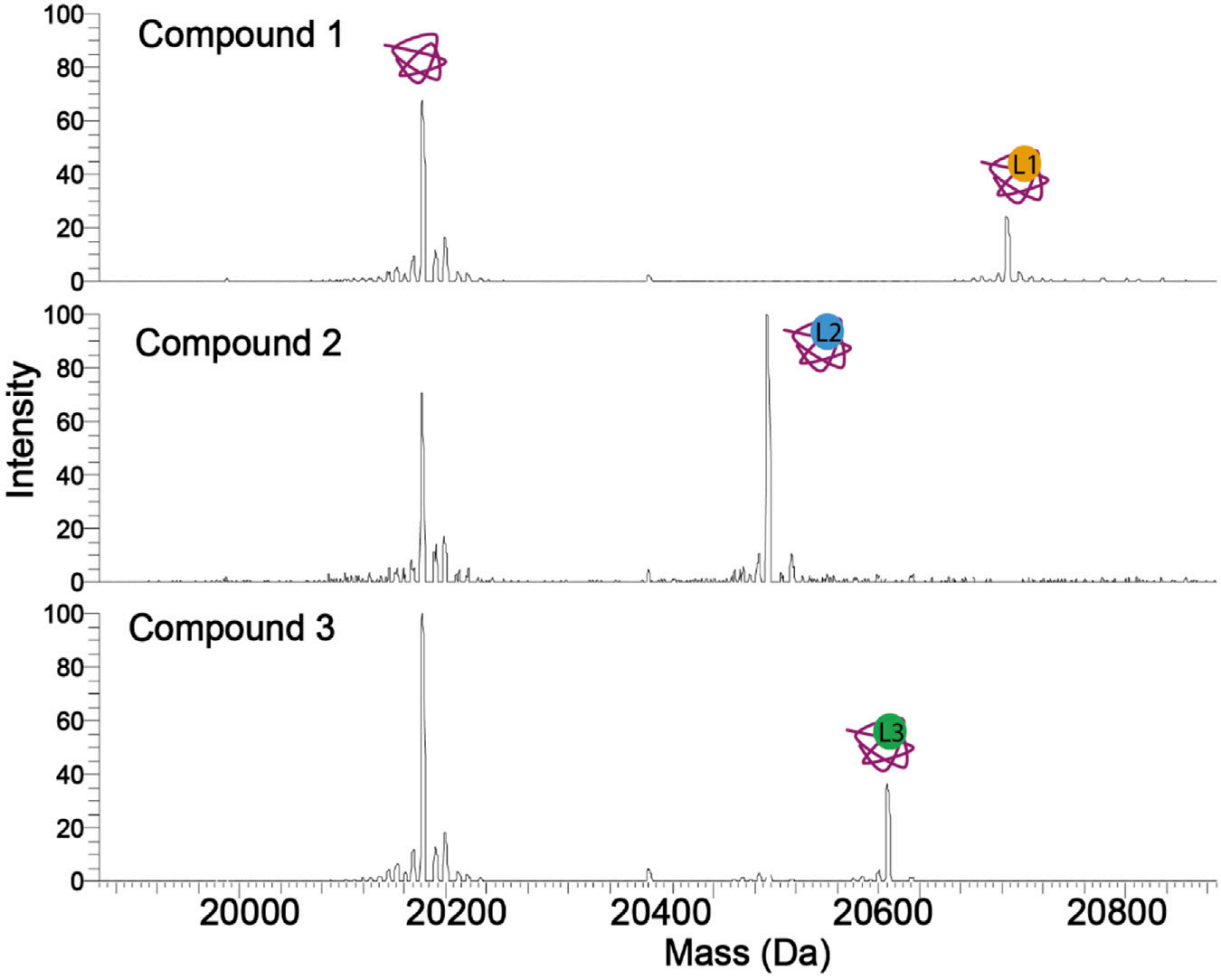
Spectra of a protein in complex with three compounds (ligands). The tighter binding compound yields a more intense complex peak. Therefore, without calculating the K_d_, the affinity of a compound against a protein can be estimated. Compound 2 has the highest occupancy to the protein, followed by compounds 3 and 1.

The typical native MS analysis for drug-binding requires only 1–2 μl of protein of interest at low-micromolar concentration. Given the broad dynamic range of signal detection and uniform ionization efficiencies between protein and protein-drug complexes (Peschke et al., 2004; Mehmood et al., 2015), drug-binding affinity can be unbiasedly determined. By looking directly at the spectra (Figure 2), the affinity of the protein-ligand interaction can be quickly evaluated without further analysis. The intensity of the peak corresponding to the protein-drug complex will increase with increasing protein-drug affinity.

Quantitative measurement of affinities requires the measurement of the drug K_d_, which can be performed by the titration approach, studied by Daniel et al. (Daniel et al., 2002), and fitting the data to the equation:

$$\frac{I\left(\mathrm{PL}\right)}{I\left(P\right)}=\frac{1}{2}\left(-1-\frac{{\left[P\right]}_{0}}{K_{d}}+\frac{{\left[L\right]}_{0}}{K_{d}}+\sqrt{4\frac{{\left[L\right]}_{0}}{K_{d}}+{\left(\frac{{\left[L\right]}_{0}}{K_{d}}-\frac{{\left[P\right]}_{0}}{K_{d}}-1\right)}^{2}}\right)$$

**FIGURE 3:**
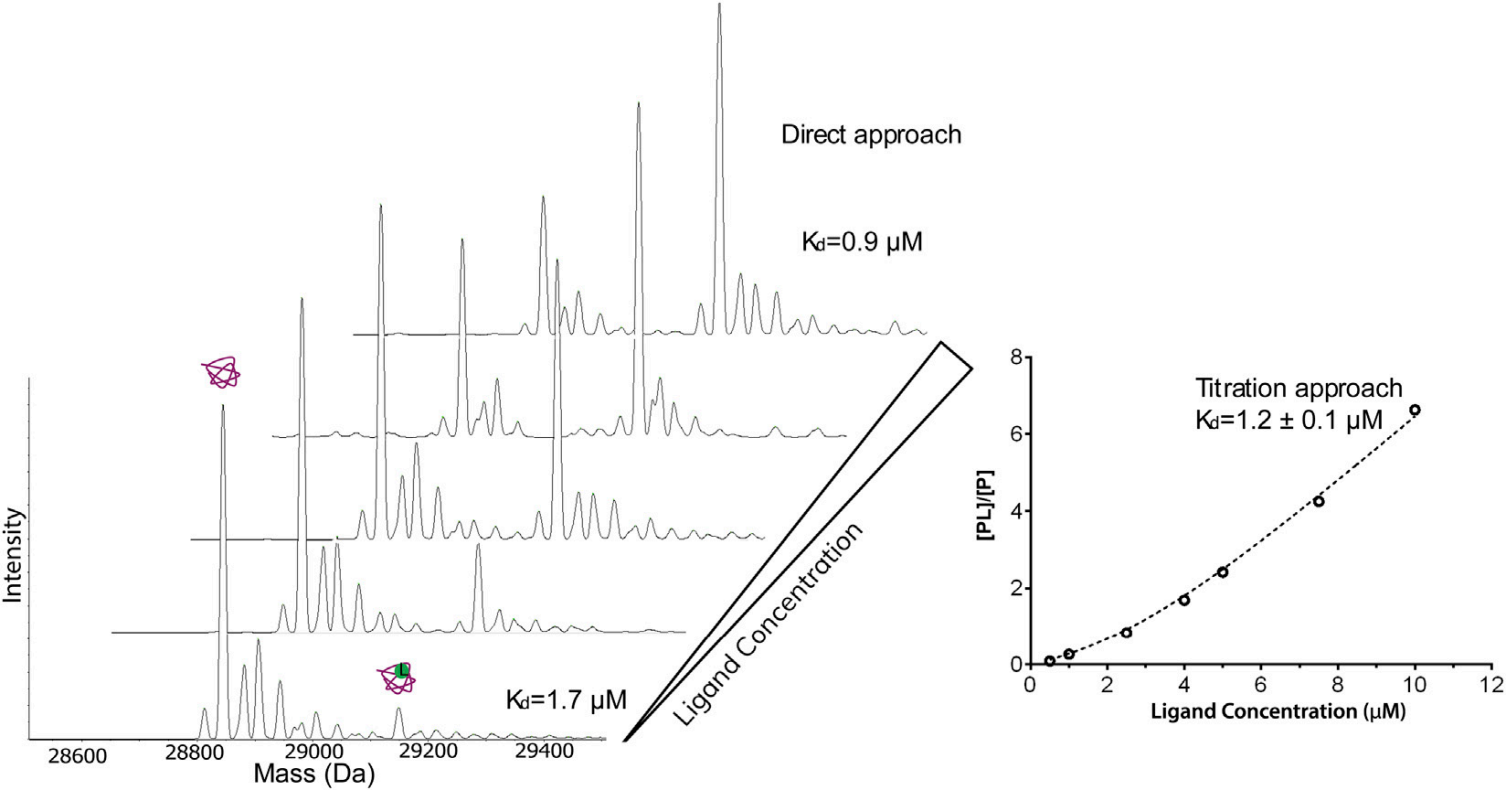
Native ESI-MS spectra and the fitted curve of titration of dichlorphenamide against 4 μM carbonic anhydrase I in 50 mM ammonium acetate, 2% dimethylsulfoxide. The concentration of dichlorphenamide was varied. The measured K_d_ from the titration curve was 1.2 μΜ which is in good agreement with the values calculated based on a single spectrum using the direct approach (1.7 and 0.9 μΜ) and published values.

A different approach was applied by Liu et al. to quantify the interactions between bovine b-lactoglobulin and a series of fatty acids by direct ESI-MS assay (Bagal et al., 2009; Liu et al., 2011). The equation of this assay is:

$$K_{a}=\frac{1}{K_{d}}=\frac{R}{{\left[L\right]}_{0}-\frac{R}{1+R}{\left[P\right]}_{0}},R=\frac{I\left(\mathrm{PL}\right)}{I\left(P\right)}$$

where I (PL) is the complex and I(P) the apoprotein peak intensity, respectively, R is their ratio, [P]_0_ and [L]_0_ are the concentrations of the protein and the ligand, and K_a_ is the association constant. Both approaches provide consistent results (Figure 3). However, the latter approach requires only a single concentration point for K_d_ measurement. Therefore, due to its speed and low material consumption, native MS has the potential to be the primary choice to acquire the affinity information of drug-binding in high throughput screening as it has been shown in various studies discussed in *High-Definition Screening by Native MS* (Maple et al., 2012; Woods et al., 2016; Nguyen et al., 2021). This is a unique feature of native MS compared to other biophysical methods. The dissociation constants of thousands of compounds against a protein target can be reliably ranked using this assay, which will mandate the selection of those that will move to the next step of the screening process.

**FIGURE 4:**
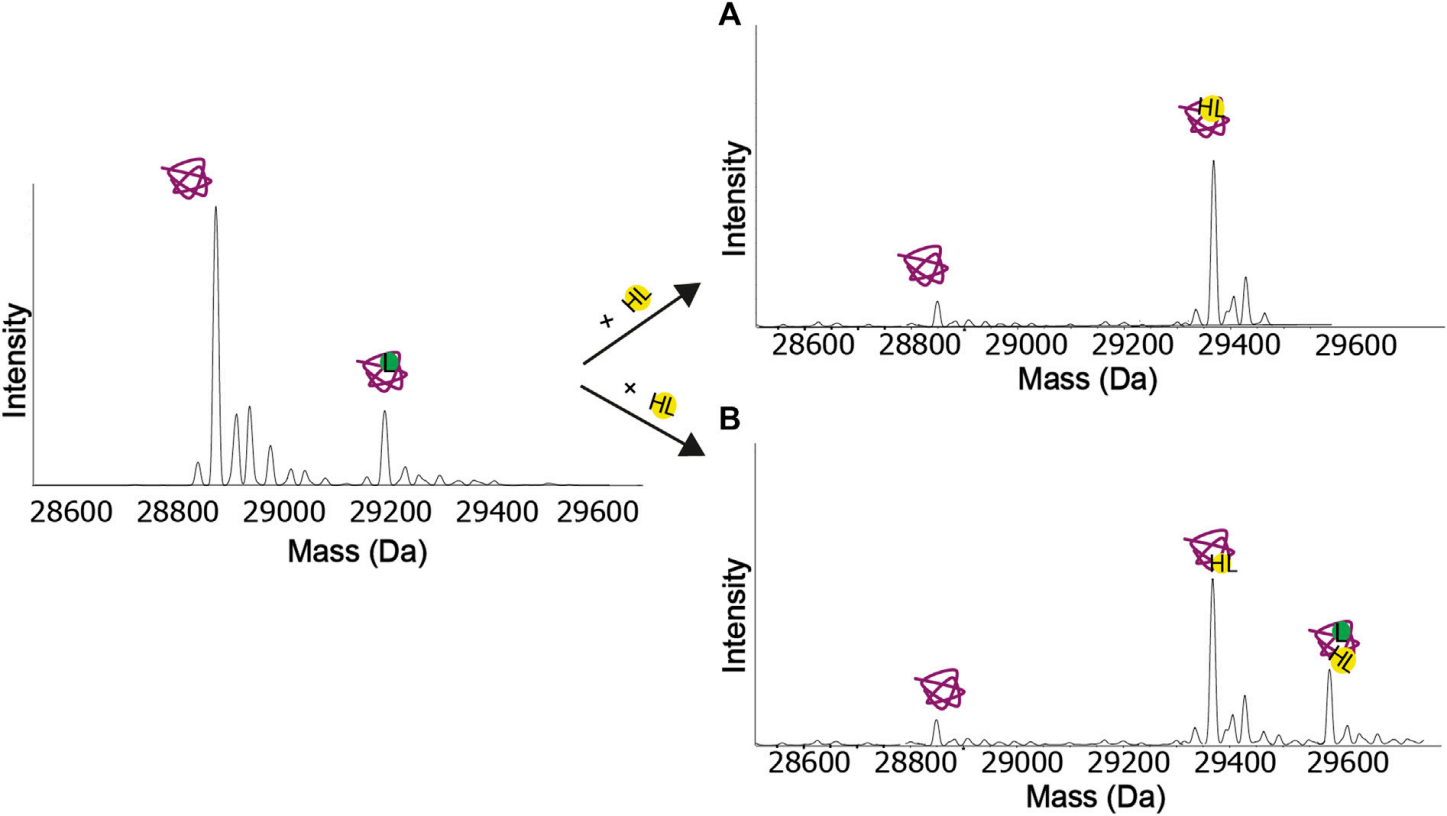
Competition experiments. **(A)** Both the ligand (L) and the hot ligand (HL) bind at the same binding site. When the concentration of the hot ligand increases, the protein-ligand peak eventually disappears. **(B)** The ligand and the hot ligand do not bind at the same binding site. When the concentration of the hot ligand increases and the protein gets saturated, the protein-ligand peak eventually disappears, and a new peak appears, corresponding to the protein with both ligands bound.

The ion intensity ratio between the free protein and the protein−ligand complex is measured for affinity measurements, and ideally, the target protein concentration should be below the expected K_d_ value. If the protein concentration is very low, such as that it appears in the noise level of the spectrum, the measurement of the intensity of the peaks may be unreliable. Therefore, using a protein concentration that will yield a high-intensity protein peak is crucial during a screening experiment, and that will allow ranking the affinities of the compounds against that protein reliably. Moreover, native MS experiments can be configured in a competitive format. Competition among different binding partners of a target molecule for its binding site can provide information about the binding affinities and specificity of host–guest complexes binding. For example, if a known ligand (hot ligand, HL) with a known binding site is available, this can be used to quickly assess the specificity for that site for a different ligand (L). Native MS competitive binding experiments consist in keeping the protein and L concentrations constant, while increasing the HL concentrations. The relative abundances of the different species in the mass spectra allow assessing whether the displacement of L with HL is competitive or not. If the HL is competing with the L for the same site, the fraction of the complex with the L decreases and the fraction of the complex with the HL increases as the HL concentration is increased as shown in Figure 4A. If the HL is not binding in the same site as the L, as the HL concentration increases and the protein becomes saturated, the peak of the protein with the L will decrease, and an additional HL + L peak will appear (Figure 4B). Jørgensen et al. (1998) used the competition approach to study the specificity of the interactions between the glycopeptide antibiotics (vancomycin, ristocetin) and several peptide and also measure their affinities. Wortmann et al. (2008), in a study of kinase inhibitors, followed the disappearance of the peak of a test ligand relative to a reference ligand in the low *m/z* range of the mass spectrometer, allowing them to determine high-affinity binding constants in the picomolar range.

Additional to affinity measurements, biomolecules’ thermodynamic and kinetic properties can be investigated by following the abundance of different species as a function of temperature and over a time course (Gülbakan et al., 2015). Thermodynamics are measured using custom variable-temperature ESI sources that enable precise temperature control of the analyte solution prior to ion formation.

Cong et al. (2016) investigated the thermodynamics of lipid binding to AmtB, an integral membrane protein of *Escherichia coli*. Their approach allowed them to determine the thermodynamics of individual binding events for lipids with variable chain lengths, resolving unique thermodynamic properties. Another study by Marchand et al. (2018) determined the entropic and enthalpic contributions to the binding equilibrium of G-quadruplex nucleic acid structures and their ligands using a temperature-controlled nano-ESI (Wilm and Mann, 1994) source. The Klassen group measured biotin’s dissociation rate (k_off_) from the tetrameric protein streptavidin (Deng et al., 2013). Marchand et al. (2020) in 2020 developed a temperature-jump electrospray source for mass spectrometry that allows performing fast kinetics experiments (0.16–32 s) at different temperatures (10–90°C).

### Membrane Proteins

There are several analytical challenges in studying membrane proteins because of the complex interactions and environmental constraints that accompany their amphipathic nature. The natural abundance of membrane proteins is typically low, and overexpression and purification of membrane proteins in high yields can be challenging (Wagner et al., 2007; Zweers et al., 2009; Gubellini et al., 2011).

Native MS has become an emerging technique in recent years for investigating the structure, dynamics, and interactions of membrane proteins (Barrera et al., 2009; Laganowsky et al., 2013; Gupta et al., 2018). The sensitivity and resolution of native MS render it a powerful tool to investigate the membrane proteins and various aspects of their structure and function, such as macromolecular assemblies, lipid/ligand interactions, post-translational modifications and, most importantly, the interplay between them. Usually, membrane proteins are studied in their natural membrane or encapsulated in a membrane mimetic to solubilize the protein prior to analysis (Strop and Brunger, 2005). Different reconstitution systems, such as detergents (Laganowsky et al., 2013), nanodiscs (Marty et al., 2016) and amphipathic polymers or amphipols (Calabrese et al., 2015) have been tested and sometimes proved critical in preserving subunit and lipid interactions of membrane proteins (Hopper et al., 2013).

**FIGURE 5:**
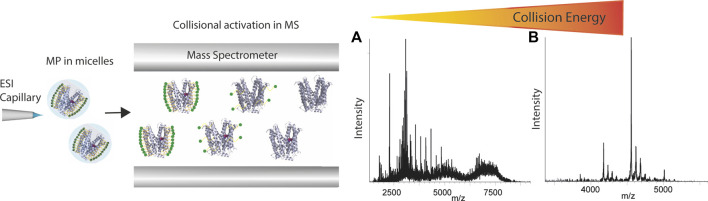
Schematic representation of how membrane proteins are studied using native mass spectrometer. Proteins are first desolvated and ionized, and then the micelle is removed inside the mass spectrometer applying high energies. The spectra show that with increasing collisional energy applied in the mass spectrometer, the spectrum can go from **(A)** micelle cluster dominated to **(B)** clear peaks representing the membrane protein.

Native MS has been applied for analyzing various membrane mimetics, which cause significant effects on the quality of mass spectra. For example, detergent micelles cause a huge mass heterogeneity which hampers the accurate mass measurement of membrane proteins, and therefore detergent adducts need to be removed by activation processes in the mass spectrometer (Figure 5). The activation energy applied may cause protein unfolding and disruption of ligand interactions in the gas-phase, hence care must be devoted to selecting a detergent which is able to preserve the native state of the membrane protein in solution and optimize the quality of mass spectra. It has been shown that the chemical properties of the detergents mediate the charge state, both during ionization and detergent removal in the mass spectrometer (Reading et al., 2015). Therefore, screening of different detergents may be required to find a suitable one (Yen et al., 2017). Various studies have shown the capability of native MS in interrogating membrane protein interactions. Binding and affinity measurement of small-molecules to membrane proteins has been investigated for the first time in the Robinson group (Marcoux et al., 2013). In a 2013, the strength of the interaction between a 17-residue peptide and the homotrimeric *E. coli* outer membrane porin F (OmpF) was quantified *via* the titration approach (Housden et al., 2013) which enabled the observation of OmpF bound to up to three peptides. The resulting K_d_ value agreed with that derived from ITC. In the same year, measuring the rates of lipid binding and calculating the K_d_ values showed that the ATP-binding cassette transporter P-glycoprotein binds diacylglycerides more tightly than zwitterionic lipids (Marcoux et al., 2013). The application of native MS has also been demonstrated for GPCRs, one of the most important protein families for drug discovery. In a study by Hsin-Yung Yen et al. (2017), the ligand/drug-binding of a human purinergic receptor P2Y1R can be preserved in a high-resolution mass spectrometer. Intriguingly, the resolution of mass spectrometry revealed the impact of receptor phosphorylation on attenuating the binding of the drug MRS2500 but not that of ATP. The importance of solution conditions in maintaining native GPCR interactions in MS has recently been highlighted by an investigation into the effects of nonvolatile salts on GPCR−small molecule interactions (Agasid et al., 2021). The inclusion of Tris and NaCl in the ESI solution was demonstrated to significantly strengthen the noncovalent interaction of the receptor to the endogenous ligand glucagon. In addition to ligand interactions of GPCRs, Gavriilidou et al. (2019) characterized turkey β1 adrenergic receptor in complex with mini-Gs, an engineered G_αs_ subunit, and the impact of ligands on complex stability. A full agonist (isoprenaline) stabilizes complex formation whereas an inverse agonist (S32212) disrupts the receptor-mini-Gs complex, showing the potential of native MS in investigating the effect of small molecules on GPCR coupling. By exploring these effects with experimental GPCR-targeting drugs, it may be possible to examine bias toward particular signaling pathways, thereby facilitating the development of highly selective therapeutic agents. Native MS has shown a significant advantage in studying membrane protein-lipid interactions. The resolution of this technique enables the detailed interrogations of a wide range of membrane proteins bound to different lipids, and the impact of acyl chain lengths and degrees of saturation on lipid-binding (Gault et al., 2016). In 2014, Bechara et al. (2015) published a protocol for identifying lipids to ABC transporter TmrAB *via* a progressive delipidation approach. By controlling the extent of protein delipidation *via* timed exposure to detergent, the preferential binding of TmrAB to negatively charged phosphatidylglycerol was revealed, and the potential role of lipids in modulating glycolipid translocation of TmrAB was proposed. In a study by the Robinson group, the specific effect of phosphatidylinositol-4,5-bisphosphate PIP_2_ on G protein-coupling of three class A GPCRs was unveiled (Yen et al., 2018). The native MS results showed that PIP_2_ significantly increased mini-Gs’ coupling to the β1 adrenergic receptor, whereas other phospholipids did not possess a similar effect. Joint use of native MS, computational modelling and site-directed mutagenesis enabled elucidation of the mechanism of PIP_2_ in bridging the receptor and the G proteins, highlighting the structural specificity of this phenomenon. A recently developed approach, nativeomics (Gault et al., 2020), aims at combining native MS with small-molecule fragmentation to directly identify bound molecules ejected after native MS. The mass spectrometer is optimized and fine-tuned in detecting both intact protein−ligand complexes at the high *m/z* range and fragmented ligands at the low *m/z* range. The strength of this approach is that it enables determination of the chemical identity of endogenous ligands or lipids and drugs of unknown identity bound to membrane proteins. In order to achieve this, multiple rounds of fragmentation are applied to progressively dissociate the protein−ligand assembly and yield ligand partners for fragmentation.

A revolutionary new method that allows membrane proteins to be directly studied from native membranes was recently developed by Robinson’s group and was named SoLVe-MS (sonication of lipid Vesicles for MS) (Chorev et al., 2018). To enable MS analysis, large membrane fragments isolated from cells are sonicated to produce smaller liposomes. This technique provided a breakthrough in studying endogenously expressed membrane proteins and their associations with native environment such as lipids and ligands, without purifying the proteins.

## High-Definition Screening by Native MS

The application of native MS to compound library screening has only recently emerged, as outlined in the following examples. The native MS screening approach shows a particular potential for fragment-based drug discovery (FBDD). The ability to capture the weak binding of fragments at a millimolar range of affinity and the low sample consumption widens the accessibility even for compounds with poor solubility (Vivat Hannah et al., 2010; Woods et al., 2016; Gavriilidou et al., 2018b). In order to improve the throughput, an automated electrospray platform, the NanoMate, was introduced (Zhang et al., 2003). This chip-based nano-ESI (Wilm and Mann, 1994) system provides consistent electrospray conditions across each analytical run, improving reproducibility. NanoMate automated sampling also significantly increases the analytical throughput compared to manual sample manipulation. The proof-of-principle study of Maple et al. has demonstrated the native MS screening of a fragment library consisting of 157 compounds against an apoptotic protein target within 6 h, using the NanoMate system (Maple et al., 2012). The throughput and results are comparable to those that NMR or ITC-based library screening approaches can obtain. In another study by Woods et al., a 720-member fragment library was screened, and ESI-MS affinity measurements correlated with the ones obtained from SPR when followed-up by X-ray crystallography (Woods et al., 2016). A recent study showed protein−small molecule interactions from mixtures containing up to ∼8,900 potential small molecule ligands in a single manual measurement using nano-ESI (Wilm and Mann, 1994) emitters in combination with a rapid, low-volume gel filtration step to remove unbound molecules (Nguyen et al., 2021).

**FIGURE 6:**
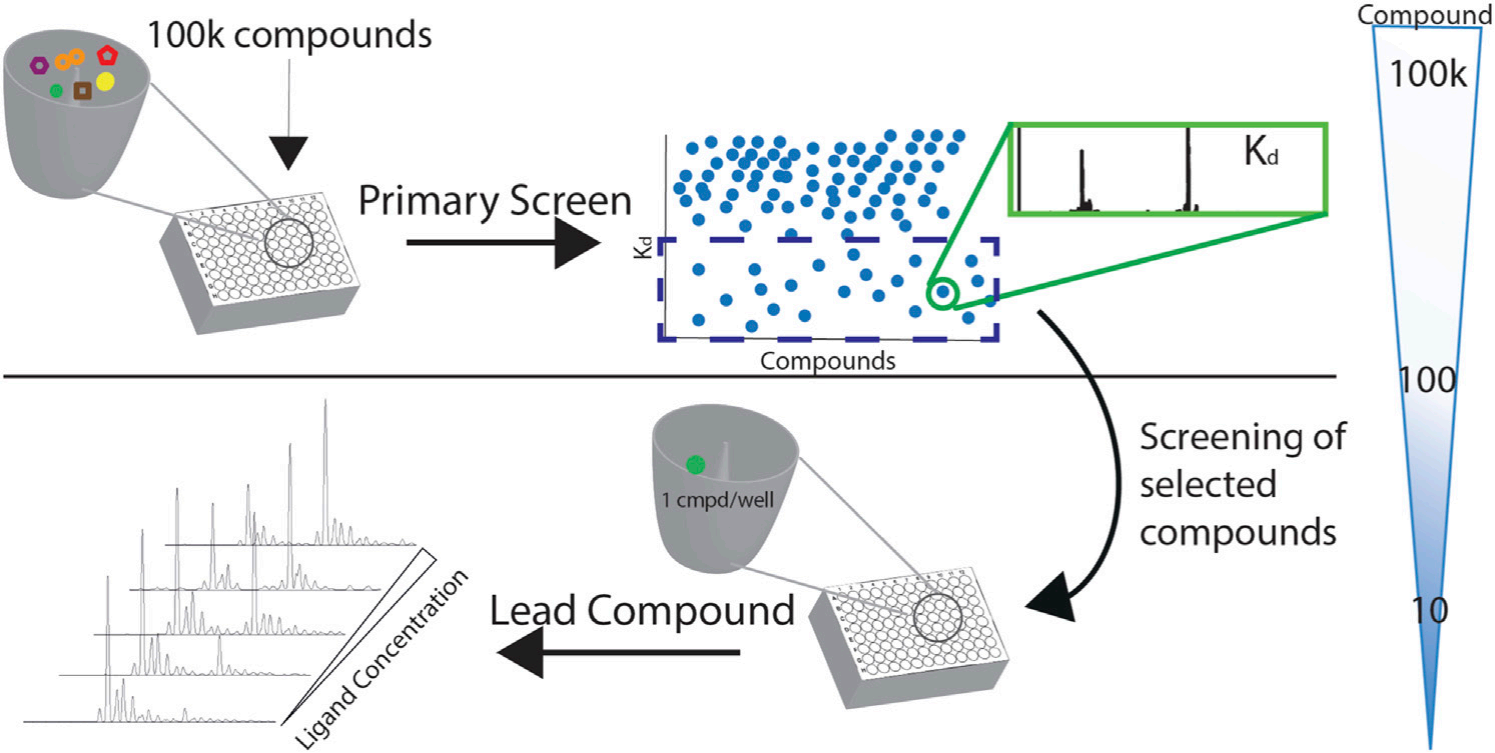
A screening cascade with native MS. The compounds of the library of the primary screen are distributed in 384-format wells. K_d_s of the complexes are measured with the direct approach, and the compounds of desirable affinity are chosen for further validation and screening as singletons.

Screening with native MS increases the throughput by allowing drug multiplexing per well. The sample consumption is very low compared to other methods and has a wide dynamic range. The number of compounds per well is mandated by the resolution needed to separate protein complexes with compounds with a slight mass difference. With the current technology and using multiplexed libraries, it is possible to screen over 50 k compounds in less than 8 h. At the end of the screening, a list of compounds and their affinities to the protein is generated. Based on the K_d_ values, compounds with the desirable affinity, for example, K_d_<50 μM, are chosen for further validation. The validation will include the specificity of binding to the protein-target by screening these compounds to a different protein or in a competition format by using a hot ligand. Moreover, the K_d_ values measured with the direct approach are confirmed with the titration method (Figure 6).

Native MS is a very advantageous method during screening due to its supreme speed, selectivity, sensitivity, and quantitative capability.

**FIGURE 7:**
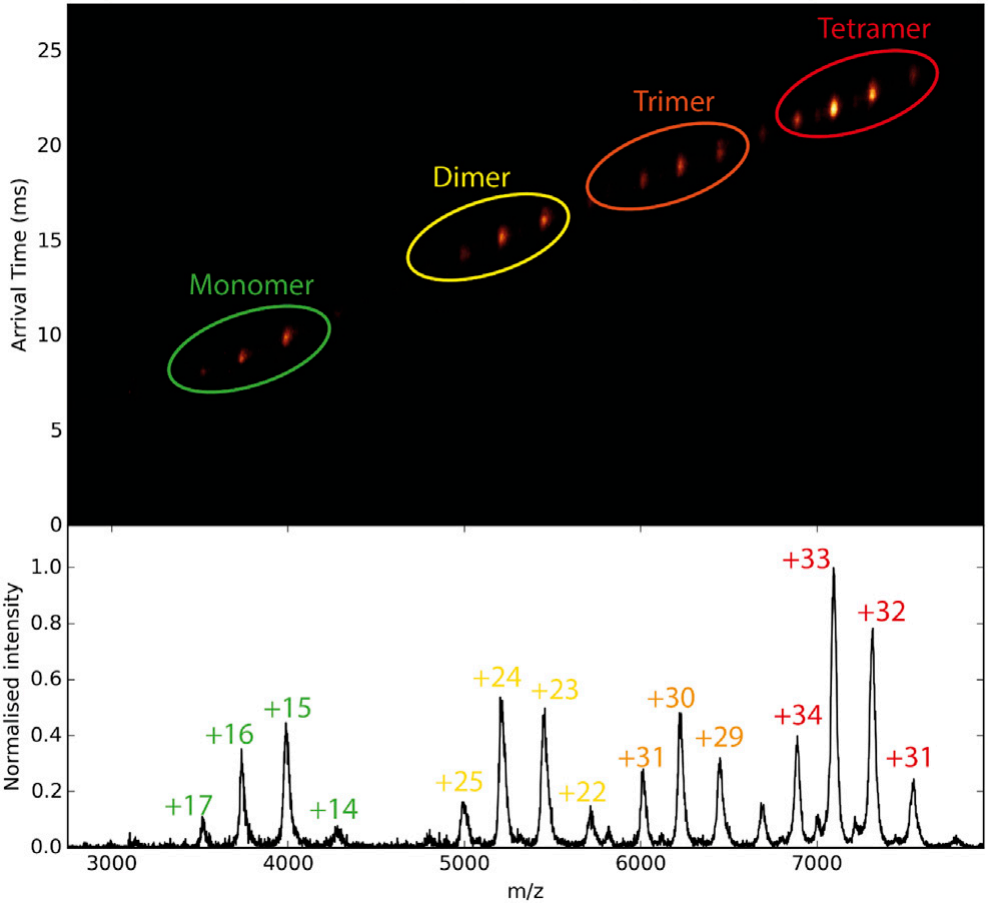
Schematic diagram of ion mobility data. In the middle, the mobilogram is shown where the drift time and *m/z* are combined in 2D representation. With the increasing mass of the protein, the drift time of each species increases.

Furthermore, mass spectrometry combined with ion mobility can reveal conformational changes of proteins upon binding to small molecules. This is a unique advantage over the other biophysical methods, as in a single ion mobility MS experiment, the binding affinity and the conformational impact of a compound to a protein can be measured. Ion mobility spectrometry (IMS) (Zhong et al., 2012) is the analytical technique that separates gas-phase ions based on their different mobility through a buffer gas at an applied electric field. The mobility of each ion in the gas will define its travel or drift time (Figure 7). The measured drift time is proportional to the collisional cross-section (CCS) of the ion, which is a physical property of the molecule and depends on its shape. Once the CCS is determined, it can be related to its quaternary structure (Ruotolo et al., 2008; Bush et al., 2010; Jurneczko and Barran, 2011; Lanucara et al., 2014). In contrast to MS, the separation of gas-phase ions in IMS is based on charge and shape rather than their *m/z* ratio.

The combination of IMS with MS, commonly referred to as IM-MS, affords 2D separation of analytes on the size–to–charge and mass–to–charge axes, respectively (Figure 7). Several studies on highly complex mixtures have shown that IM–MS gives far greater resolution and information than is possible by either method alone (Ruotolo et al., 2007; Duijn et al., 2009; Hopper and Oldham, 2009; Smith et al., 2010; Bleiholder et al., 2011; Bleiholder et al., 2013). Studies to investigate changes in protein structure that are driven by ligand binding have been carried out. For example, Lai et al. used native IM-MS to characterize large-scale conformational shifts of the *Escherichia coli* molecular chaperone DnaK in response to nucleotide and substrate binding. Unique conformational states that arose due to the allosteric effects of small molecule interactions were identified and combined with results from double electron−electron resonance spectroscopy to confirm structural data derived from both NMR and X-crystallography experiments (Lai et al., 2017). Ashcroft’s group screened small molecule inhibitors against amyloid precursors, identifying the interacting protein species and defining the mode of inhibition. They were able to classify a variety of small molecules that are potential inhibitors of human islet amyloid polypeptide (hIAPP) aggregation or amyloid-beta 1–40 aggregation as specific, nonspecific, colloidal or non-interacting (Young et al., 2014). Small conformational changes in globular proteins that occur upon ligand binding can also be observed through slight changes in the arrival time distribution (Atmanene et al., 2012; Stojko et al., 2015). The Klassen group has combined multistage ion activation and IM to determine the identity of bound ligands during a screening, an approach they have termed “Catch-and-Release” (CaR) ESI-MS (El-Hawiet et al., 2012; Rezaei Darestani et al., 2016; El-Hawiet et al., 2018). In this approach, a protein of interest is mixed with a library of ligands and the resulting mixture is analyzed by native MS. Multiple stages of ion activation and fragmentation are applied, in which the ligands are fragmented. By combining this process with IM, both the arrival time distributions and the fragmentations patterns of the ligands can be used to ascertain their identity.

Collision induced unfolding (CIU) is a collisional activation method in which the unfolding of protein complexes in the gas-phase is monitored with IM-MS. During IM-MS screening, CIU fingerprints can be uniquely related to specific protein-ligand binding modes (Hopper and Oldham, 2009; Hyung et al., 2009; Niu et al., 2013). The unfolding pathway of the protein can be followed in detail to allow the comparison between different conformational families of the protein (Han et al., 2011; Han et al., 2012; Han and Ruotolo, 2013). Ruotolo’s group developed a data analysis workflow to remove chemical noise patterns caused by ionized surfactants during studies of membrane proteins (Fantin et al., 2019). Following the denoising protocol, separate gas-phase unfolding signatures with CIU for lipid and protoporphyrin binding to the dimer of translocator protein (TSPO) were generated. Complexes containing ligands known to bind at two separate sites were detected as possessing differential stabilities using CIU, where protoporphyrin IX binding provided a greater degree of gas-phase stabilization for TSPO than any lipids assessed. These data were combined with liquid−liquid extracts to propose and identity unknown endogenous TSPO ligands.

It is expected that as the resolution of IM-MS methods in both the mobility and *m/z* dimensions and the accuracy of computational models of protein structure and dynamics increase the use of IM-MS for studying the impact of ligand binding on protein structures and assemblies will also significantly increase.

## Future Perspective of Native MS in Structure-Based Drug Leads Discovery

Technological advances continue to emerge, and the recent introduction of orbitrap mass analyzers modified for protein assemblies affords excellent resolution and sensitivity, enabling facile definition of concurrent binding of small molecules in a MDa complex (Rose et al., 2012; Gault et al., 2016). Taking advantage of the high resolution, native MS is pacing towards the analysis of proteins and protein complexes directly from cells, characterizing their heterogeneity and flexibility in real time (Gan et al., 2017; Chorev et al., 2018). Intact assemblies from membranes, without chemical disruption, can be analyzed using mass spectrometry to define their composition and characterize any endogenous ligand or lipid binding. This development is analogous to the Cryo-EM structure determination of MDa complexes from native cell extracts (Kyrilis et al., 2021). However, improvements in data analysis are still required for this field to bloom, as the spectra generated from these studies can be difficult and time-consuming to annotate. The improvement of data analysis software will also allow native MS to be integrated as a routine method in the pharmaceutical sector.

The current mass spectrometers allow high throughput screening of small molecules for binding against a protein target and determining the dissociation constant. As shown and discussed above, the thermodynamic and kinetic properties of biomolecules can also be measured with MS using modified instrumentation (Gülbakan et al., 2015). Therefore, the commercialization of these novel sources for mass spectrometers that will vary the temperature in a controlled way over a time course will allow for the detailed thermodynamic study of protein-ligand complex.

3D models of macromolecular complexes have been deduced by combining MS experiments with data from crystallography, NMR, small-angle X-ray scattering and EM (Lebrette et al., 2015; Nematollahi et al., 2015) and it is expected that in the future more structures will be solved with the help of mass spectrometry. Structure-based drug discovery (SBBD) has also benefited from the introduction of mass spectrometry as a complementary tool accelerating the sample screening workflow (Olinares et al., 2021). Based on a combinatorial study of native MS and other techniques, Heck’s group (Snijder et al., 2017) revealed details of the interactions between the Kai proteins, a system that cyanobacteria use as a circadian oscillator. The stoichiometry of the different Kai proteins was monitored by native MS, allowing for structural characterization by single-particle cryo-electron microscopy (cryo-EM) and MS. Pseudoatomic models of biomolecular assemblies have been generated with computational methods based on data from native mass spectrometry (Marklund and Benesch, 2019). Politis et al. show many examples implementing this approach (Politis et al., 2014). They describe a method for the characterization of protein assemblies structures integrating results derived from different MS-based techniques with modeling data. They encoded results from native MS, bottom-up proteomics, IM–MS and chemical cross-linking MS into modeling restraints to compute the most likely structures. Esser et al. (2021) presented native electrospray ion-beam deposition (native ES-IBD) for the preparation of extremely high-purity cryo-EM samples, based on mass selection in the gas-phase. Folded protein ions generated by native MS were mass-filtered with fine tuning of the mass spectrometer, and gently deposited on cryo-EM grids, and subsequently frozen in liquid nitrogen. Single particle analysis revealed that they remain structurally intact.

Notably, native MS allows the identification of a protein bound ligand when it is featureless in cryo-EM and X-ray maps. In fact, in the specific case of membrane proteins, known to be valuable therapeutic targets, bound detergents or lipids cannot be identified completely by cryo-EM and X-ray crystallography unless their hydrophobic tails are constrained in specific places. Conversely, the hydrophilic heads tend to be defined more accurately due to the electrostatic interactions with the protein partner. Moreover, multiple chemical species bound to membrane proteins, such as a mixture of lipids present at partial occupancy, produce poorly defined electron or cryo-EM densities, making it hard to assign chemical entities. Native MS can resolve lipid mixture bound to protein and provide the relative abundance of each component. Additionally, large multimeric targets could lose ancillary or weakly attached subunits when extracted and purified from their native environment to perform SBBD studies. We envisage that in the not-too-distant future, *in situ* native MS will add another dimension to the 3D views of large macromolecular assemblies currently imaged in their native frozen-hydrated state by electron cryotomography (ECT) (Oikonomou and Jensen, 2017). The so-called mass spectrometry imaging (MSI) (Griffiths et al., 2019) will offer spatial information about drug distribution directly at the cellular level, evaluation of druggability *in situ*, and provide crucial insights about the ripple effects of a drug candidate on whole cellular physiology speeding up the development of a drug considerably.

## Conclusion

In the last few years, native MS has become a well-established technique for drug discovery. Its high sensitivity, simplicity, speed, wide dynamic range, low protein and ligand consumption, and the possibility of automation and high throughput makes it an integral component of the biophysical toolkit commonly used for primary screening, adding to techniques such as NMR and SPR. Moreover, the ability to explore all the aspects of protein-ligand interactions and dynamics is the basis of the unique potential of native MS in the fragment hit identification. The majority of published applications of native MS in drug discovery are on soluble proteins. Nevertheless, in recent years the frontier conquered by native MS in membrane proteins broadens the repertoire of protein targets screened by this technique. K_d_ determinations by direct-ESI assay or titration experiments have provided accurate values and can be used to quickly assess the compounds’ affinity to the target protein during a screening campaign. Native MS can also efficiently assess compound specificity for a particular binding site in competitive binding experiments. It is possible to distinguish multiple binding sites with the appropriate instrumental parameters, revealing complex allosteric mechanisms. In addition, IMS studies during screening have provided insight into conformational changes of a protein upon binding to a compound. The continuous improvements in mass spectrometry hardware and software are expanding the limits of native MS applications. In addition, native MS is expected to be fully integrated with other structural biology techniques, such as X-ray crystallography, cryo-EM, and in a not distant future cryo-ET, in the drug discovery pipeline, providing unprecedented insights on protein-ligand binding and ligands screening that could significantly impact the drug discovery process.

## References

[B1] Aebersold R. Mann M. (2016). Mass-spectrometric Exploration of Proteome Structure and Function. Nature 537 (7620), 347–355. 10.1038/nature19949 27629641

[B2] Agasid M. T. Sørensen L. Urner L. H. Yan J. Robinson C. V. (2021). The Effects of Sodium Ions on Ligand Binding and Conformational States of G Protein-Coupled Receptors-Insights from Mass Spectrometry. J. Am. Chem. Soc. 143 (11), 4085–4089. 10.1021/jacs.0c11837 33711230 PMC7995251

[B3] Atmanene C. Petiot-Bécard S. Zeyer D. Van Dorsselaer A. Vivat Hannah V. Sanglier-Cianférani S. (2012). Exploring Key Parameters to Detect Subtle Ligand-Induced Protein Conformational Changes Using Traveling Wave Ion Mobility Mass Spectrometry. Anal. Chem. 84 (11), 4703–4710. 10.1021/ac203223h 22533353

[B4] Badman E. R. Myung S. Clemmer D. E. (2005). Evidence for Unfolding and Refolding of Gas-phase Cytochrome C Ions in a Paul Trap. J. Am. Soc. Mass. Spectrom. 16 (9), 1493–1497. 10.1016/j.jasms.2005.04.013 16019223

[B5] Bagal D. Kitova E. N. Liu L. El-Hawiet A. Schnier P. D. Klassen J. S. (2009). Gas Phase Stabilization of Noncovalent Protein Complexes Formed by Electrospray Ionization. Anal. Chem. 81 (18), 7801–7806. 10.1021/ac900611a 19746998

[B6] Barrera N. P. Isaacson S. C. Zhou M. Bavro V. N. Welch A. Schaedler T. A. (2009). Mass Spectrometry of Membrane Transporters Reveals Subunit Stoichiometry and Interactions. Nat. Methods 6 (8), 585–587. 10.1038/nmeth.1347 19578383 PMC4066579

[B7] Bechara C. Nöll A. Morgner N. Degiacomi M. T. Tampé R. Robinson C. V. (2015). A Subset of Annular Lipids Is Linked to the Flippase Activity of an ABC Transporter. Nat. Chem 7 (3), 255–262. 10.1038/nchem.2172 25698336

[B8] Benesch J. L. Ruotolo B. T. (2011). Mass Spectrometry: Come of Age for Structural and Dynamical Biology. Curr. Opin. Struct. Biol. 21 (5), 641–649. 10.1016/j.sbi.2011.08.002 21880480 PMC3193349

[B9] Benkestock K. Sundqvist G. Edlund P.-O. Roeraade J. (2004). Influence of Droplet Size, Capillary-Cone Distance and Selected Instrumental Parameters for the Analysis of Noncovalent Protein-Ligand Complexes by Nano-Electrospray Ionization Mass Spectrometry. J. Mass. Spectrom. 39 (9), 1059–1067. 10.1002/jms.685 15386746

[B10] Bernstein S. L. Dupuis N. F. Lazo N. D. Wyttenbach T. Condron M. M. Bitan G. (2009). Amyloid-β Protein Oligomerization and the Importance of Tetramers and Dodecamers in the Aetiology of Alzheimer's Disease. Nat. Chem 1 (4), 326–331. 10.1038/nchem.247 20703363 PMC2918915

[B11] Bleiholder C. Do T. D. Wu C. Economou N. J. Bernstein S. S. Buratto S. K. (2013). Ion Mobility Spectrometry Reveals the Mechanism of Amyloid Formation of Aβ(25-35) and its Modulation by Inhibitors at the Molecular Level: Epigallocatechin Gallate and Scyllo-Inositol. J. Am. Chem. Soc. 135 (45), 16926–16937. 10.1021/ja406197f 24131107

[B12] Bleiholder C. Dupuis N. F. Wyttenbach T. Bowers M. T. (2011). Ion Mobility-Mass Spectrometry Reveals a Conformational Conversion from Random Assembly to β-sheet in Amyloid Fibril Formation. Nat. Chem 3 (2), 172–177. 10.1038/nchem.945 21258392 PMC3073516

[B13] Borysik A. J. H. Radford S. E. Ashcroft A. E. (2004). Co-populated Conformational Ensembles of β2-Microglobulin Uncovered Quantitatively by Electrospray Ionization Mass Spectrometry. J. Biol. Chem. 279 (26), 27069–27077. 10.1074/jbc.M401472200 15100226

[B14] Breuker K. Brüschweiler S. Tollinger M. (2011). Electrostatic Stabilization of a Native Protein Structure in the Gas Phase. Angew. Chem. Int. Ed. 50 (4), 873–877. 10.1002/anie.201005112 PMC3045662 21246681

[B15] Breuker K. McLafferty F. W. (2008). Stepwise Evolution of Protein Native Structure with Electrospray into the Gas Phase, 10-12 to 102 S. Proc. Natl. Acad. Sci. 105 (47), 18145–18152. 10.1073/pnas.0807005105 19033474 PMC2587555

[B16] Bush M. F. Hall Z. Giles K. Hoyes J. Robinson C. V. Ruotolo B. T. (2010). Collision Cross Sections of Proteins and Their Complexes: A Calibration Framework and Database for Gas-phase Structural Biology. Anal. Chem. 82 (22), 9557–9565. 10.1021/ac1022953 20979392

[B17] Calabrese A. N. Watkinson T. G. Henderson P. J. F. Radford S. E. Ashcroft A. E. (2015). Amphipols Outperform Dodecylmaltoside Micelles in Stabilizing Membrane Protein Structure in the Gas Phase. Anal. Chem. 87 (2), 1118–1126. 10.1021/ac5037022 25495802 PMC4636139

[B18] Chalmers M. J. Busby S. A. Pascal B. D. West G. M. Griffin P. R. (2011). Differential Hydrogen/deuterium Exchange Mass Spectrometry Analysis of Protein-Ligand Interactions. Expert Rev. Proteomics 8 (1), 43–59. 10.1586/epr.10.109 21329427 PMC3113475

[B19] Chorev D. S. Baker L. A. Wu D. Beilsten-Edmands V. Rouse S. L. Zeev-Ben-Mordehai T. (2018). Protein Assemblies Ejected Directly from Native Membranes Yield Complexes for Mass Spectrometry. Science 362 (6416), 829–834. 10.1126/science.aau0976 30442809 PMC6522346

[B20] Chowdhury S. K. Katta V. Chait B. T. (1990). Probing Conformational Changes in Proteins by Mass Spectrometry. J. Am. Chem. Soc. 112 (24), 9012–9013. 10.1021/ja00180a074

[B21] Cong X. Liu Y. Liu W. Liang X. Russell D. H. Laganowsky A. (2016). Determining Membrane Protein-Lipid Binding Thermodynamics Using Native Mass Spectrometry. J. Am. Chem. Soc. 138 (13), 4346–4349. 10.1021/jacs.6b01771 27015007

[B22] Cooper M. A. (2002). Optical Biosensors in Drug Discovery. Nat. Rev. Drug Discov. 1 (7), 515–528. 10.1038/nrd838 12120258

[B23] Daniel J. M. Friess S. D. Rajagopalan S. Wendt S. Zenobi R. (2002). Quantitative Determination of Noncovalent Binding Interactions Using Soft Ionization Mass Spectrometry. Int. J. Mass Spectrom. 216 (1), 1–27. 10.1016/s1387-3806(02)00585-7

[B24] de Azevedo W. F. Dias R. (2008). Experimental Approaches to Evaluate the Thermodynamics of Protein-Drug Interactions. Curr. Drug Targets 9 (12), 1071–1076. 10.2174/138945008786949441 19128217

[B25] Deng L. Kitova E. N. Klassen J. S. (2013). Dissociation Kinetics of the Streptavidin-Biotin Interaction Measured Using Direct Electrospray Ionization Mass Spectrometry Analysis. J. Am. Soc. Mass. Spectrom. 24 (1), 49–56. 10.1007/s13361-012-0533-5 23247970

[B26] Dobo A. Kaltashov I. A. (2001). Detection of Multiple Protein Conformational Ensembles in Solution via Deconvolution of Charge-State Distributions in ESI MS. Anal. Chem. 73 (20), 4763–4773. 10.1021/ac010713f 11681449

[B27] Douangamath A. Fearon D. Gehrtz P. Krojer T. Lukacik P. Owen C. D. (2020). Crystallographic and Electrophilic Fragment Screening of the SARS-CoV-2 Main Protease. Nat. Commun. 11, 5047. 10.1038/s41467-020-18709-w 33028810 PMC7542442

[B28] Duijn E. v. Barendregt A. Synowsky S. Versluis C. Heck A. J. R. (2009). Chaperonin Complexes Monitored by Ion Mobility Mass Spectrometry. J. Am. Chem. Soc. 131 (12), 1452–1459. 10.1021/ja8055134 19138114

[B29] Dyachenko A. Gruber R. Shimon L. Horovitz A. Sharon M. (2013). Allosteric Mechanisms Can Be Distinguished Using Structural Mass Spectrometry. Proc. Natl. Acad. Sci. 110, 7235–7239. 10.1073/pnas.1302395110/-/DCSupplemental/pnas.201302395SI.pdf 23589876 PMC3645570

[B30] El-Hawiet A. Chen Y. Shams-Ud-Doha K. Kitova E. N. Kitov P. I. Bode L. (2018). Screening Natural Libraries of Human Milk Oligosaccharides against Lectins Using CaR-ESI-MS. Analyst 143 (2), 536–548. 10.1039/C7AN01397C 29239412

[B31] El-Hawiet A. Shoemaker G. K. Daneshfar R. Kitova E. N. Klassen J. S. (2012). Applications of a Catch and Release Electrospray Ionization Mass Spectrometry Assay for Carbohydrate Library Screening. Anal. Chem. 84 (1), 50–58. 10.1021/ac202760e 22128847

[B32] Engen J. R. Wales T. E. (2015). Analytical Aspects of Hydrogen Exchange Mass Spectrometry. Annu. Rev. Anal. Chem. 8, 127–148. 10.1146/annurev-anchem-062011-143113 PMC4989240 26048552

[B33] Esser T. K. Boehning J. Fremdling P. (2021). Mass-selective and Ice-free Cryo-EM Protein Sample Preparation via Native Electrospray Ion-Beam Deposition. bioRxiv. 10.1093/pnasnexus/pgac153 PMC9802471 36714824

[B34] Fantin S. M. Parson K. F. Niu S. Liu J. Polasky D. A. Dixit S. M. (2019). Collision Induced Unfolding Classifies Ligands Bound to the Integral Membrane Translocator Protein. Anal. Chem. 91 (24), 15469–15476. 10.1021/acs.analchem.9b03208 31743004

[B35] Fenn J. B. Mann M. Meng C. K. Wong S. F. Whitehouse C. M. (1989). Electrospray Ionization for Mass Spectrometry of Large Biomolecules. Science 246, 64–71. 10.1126/science.2675315 2675315

[B36] Fenselau C. Szilágyi Z. Williams T. (2000). Intercharge Distances in Zn7-Metallothionein Analyzed by Nanospray on a Quadrupole Ion Trap and Molecular Modeling. J. Mass. Spectrom. Soc. Jpn. 48 (1), 23–25. 10.5702/massspec.48.23

[B37] Fernandez De la Mora J. (2000). Electrospray Ionization of Large Multiply Charged Species Proceeds via Dole's Charged Residue Mechanism. Analytica Chim. Acta 406 (1), 93–104. 10.1016/S0003-2670(99)00601-7

[B38] Fort K. L. van de Waterbeemd M. Boll D. Reinhardt-Szyba M. Belov M. E. Sasaki E. (2018). Expanding the Structural Analysis Capabilities on an Orbitrap-Based Mass Spectrometer for Large Macromolecular Complexes. Analyst 143 (1), 100–105. 10.1039/C7AN01629H 29138777

[B39] Gan J. Ben-nissan G. Arkind G. Tarnavsky M. Trudeau D. Noda Garcia L. (2017). Native Mass Spectrometry of Recombinant Proteins from Crude Cell Lysates. Anal. Chem. 89, 4398–4404. 10.1021/acs.analchem.7b00398 28345863 PMC5702260

[B40] Ganem B. Li Y. T. Henion J. D. (1991). Detection of Noncovalent Receptor-Ligand Complexes by Mass Spectrometry. J. Am. Chem. Soc. 113 (16), 6294–6296. 10.1021/ja00016a069

[B41] Gault J. Donlan J. A. C. Liko I. Hopper J. T. S. Gupta K. Housden N. G. (2016). High-resolution Mass Spectrometry of Small Molecules Bound to Membrane Proteins. Nat. Methods 13 (4), 333–336. 10.1038/nmeth.3771 26901650 PMC4856209

[B42] Gault J. Liko I. Landreh M. Shutin D. Bolla J. R. Jefferies D. (2020). Combining Native and 'omics' Mass Spectrometry to Identify Endogenous Ligands Bound to Membrane Proteins. Nat. Methods 17 (5), 505–508. 10.1038/s41592-020-0821-0 32371966 PMC7332344

[B43] Gavriilidou A. F. M. Gülbakan B. Zenobi R. (2015). Influence of Ammonium Acetate Concentration on Receptor-Ligand Binding Affinities Measured by Native Nano ESI-MS: A Systematic Study. Anal. Chem. 87, 10378–10384. 10.1021/acs.analchem.5b02478 26399292

[B44] Gavriilidou A. F. M. Holding F. P. Coyle J. E. Zenobi R. (2018). Application of Native ESI-MS to Characterize Interactions between Compounds Derived from Fragment-Based Discovery Campaigns and Two Pharmaceutically Relevant Proteins. SLAS DISCOVERY: Advancing Sci. Drug Discov. 23 (9), 951–959. 10.1177/2472555218775921 29852073

[B45] Gavriilidou A. F. M. Holding F. P. Mayer D. Coyle J. E. Veprintsev D. B. Zenobi R. (2018). Native Mass Spectrometry Gives Insight into the Allosteric Binding Mechanism of M2 Pyruvate Kinase to Fructose-1,6-Bisphosphate. Biochemistry 57 (11), 1685–1689. 10.1021/acs.biochem.7b01270 29499117

[B46] Gavriilidou A. F. M. Hunziker H. Mayer D. Vuckovic Z. Veprintsev D. B. Zenobi R. (2019). Insights into the Basal Activity and Activation Mechanism of the β1 Adrenergic Receptor Using Native Mass Spectrometry. J. Am. Soc. Mass. Spectrom. 30 (3), 529–537. 10.1007/s13361-018-2110-z 30511235

[B47] Geels R. B. J. Van Der Vies S. M. Heck A. J. R. Heeren R. M. A. (2006). Electron Capture Dissociation as Structural Probe for Noncovalent Gas-phase Protein Assemblies. Anal. Chem. 78 (20), 7191–7196. 10.1021/ac060960p 17037920

[B48] Grabenauer M. Wyttenbach T. Sanghera N. Slade S. E. Pinheiro T. J. T. Scrivens J. H. (2010). Conformational Stability of Syrian Hamster Prion Protein PrP(90−231). J. Am. Chem. Soc. 132 (26), 8816–8818. 10.1021/ja100243h 20536231 PMC2902166

[B49] Griffiths R. L. Sisley E. K. Lopez-Clavijo A. F. Simmonds A. L. Styles I. B. Cooper H. J. (2019). Native Mass Spectrometry Imaging of Intact Proteins and Protein Complexes in Thin Tissue Sections. Int. J. Mass Spectrom. 437, 23–29. 10.1016/j.ijms.2017.10.009

[B50] Gubellini F. Verdon G. Karpowich N. K. Luff J. D. Boël G. Gauthier N. (2011). Physiological Response to Membrane Protein Overexpression in *E. coli* . Mol. Cell Proteomics 10 (10), 007930. 10.1074/mcp.M111.007930 21719796 PMC3205863

[B51] Gülbakan B. Barylyuk K. Zenobi R. (2015). Determination of Thermodynamic and Kinetic Properties of Biomolecules by Mass Spectrometry. Curr. Opin. Biotechnol. 31, 65–72. 10.1016/j.copbio.2014.08.003 25173612

[B52] Gupta K. Li J. Liko I. Gault J. Bechara C. Wu D. (2018). Identifying Key Membrane Protein Lipid Interactions Using Mass Spectrometry. Nat. Protoc. 13 (5), 1106–1120. 10.1038/nprot.2018.014 29700483 PMC6049616

[B53] Han L. Hyung S.-J. Mayers J. J. S. Ruotolo B. T. (2011). Bound Anions Differentially Stabilize Multiprotein Complexes in the Absence of Bulk Solvent. J. Am. Chem. Soc. 133 (29), 11358–11367. 10.1021/ja203527a 21675748 PMC3140617

[B54] Han L. Hyung S.-J. Ruotolo B. T. (2012). Bound Cations Significantly Stabilize the Structure of Multiprotein Complexes in the Gas Phase. Angew. Chem. Int. Ed. 51 (23), 5692–5695. 10.1002/anie.201109127 PMC3517040 22529039

[B55] Han L. Ruotolo B. T. (2013). Traveling-wave Ion Mobility-Mass Spectrometry Reveals Additional Mechanistic Details in the Stabilization of Protein Complex Ions through Tuned Salt Additives. Int. J. Ion Mobil. Spec. 16 (1), 41–50. 10.1007/s12127-013-0121-9 PMC3607392 23539363

[B56] Heck A. J. R. van den Heuvel R. H. H. (2004). Investigation of Intact Protein Complexes by Mass Spectrometry. Mass. Spectrom. Rev. 23 (5), 368–389. 10.1002/mas.10081 15264235

[B57] Hernández H. Dziembowski A. Taverner T. Séraphin B. Robinson C. V. (2006). Subunit Architecture of Multimeric Complexes Isolated Directly from Cells. EMBO Rep. 7 (6), 605–610. 10.1038/sj.embor.7400702 16729021 PMC1479597

[B58] Hernández H. Robinson C. V. (2007). Determining the Stoichiometry and Interactions of Macromolecular Assemblies from Mass Spectrometry. Nat. Protoc. 2 (3), 715–726. 10.1038/nprot.2007.73 17406634

[B59] Hopper J. T. S. Oldham N. J. (2011). Alkali Metal Cation-Induced Destabilization of Gas-phase Protein-Ligand Complexes: Consequences and Prevention. Anal. Chem. 83 (19), 7472–7479. 10.1021/ac201686f 21863818

[B60] Hopper J. T. S. Oldham N. J. (2009). Collision Induced Unfolding of Protein Ions in the Gas Phase Studied by Ion Mobility-Mass Spectrometry: The Effect of Ligand Binding on Conformational Stability. J. Am. Soc. Mass. Spectrom. 20 (10), 1851–1858. 10.1016/j.jasms.2009.06.010 19643633

[B61] Hopper J. T. S. Yu Y. T.-C. Li D. Raymond A. Bostock M. Liko I. (2013). Detergent-free Mass Spectrometry of Membrane Protein Complexes. Nat. Methods 10 (12), 1206–1208. 10.1038/nmeth.2691 24122040 PMC3868940

[B62] Housden N. G. Hopper J. T. S. Lukoyanova N. Rodriguez-Larrea D. Wojdyla J. A. Klein A. (2013). Intrinsically Disordered Protein Threads through the Bacterial Outer-Membrane Porin OmpF. Science 340 (6140), 1570–1574. 10.1126/science.1237864 23812713 PMC3856478

[B63] Hyung S.-J. Robinson C. V. Ruotolo B. T. (2009). Gas-Phase Unfolding and Disassembly Reveals Stability Differences in Ligand-Bound Multiprotein Complexes. Chem. Biol. 16 (4), 382–390. 10.1016/j.chembiol.2009.02.008 19389624

[B64] Iribarne J. V. Thomson B. A. (1976). On the Evaporation of Small Ions from Charged Droplets. J. Chem. Phys. 64 (6), 2287. 10.1063/1.432536

[B65] Jacques M. Jeffries W. Jean-Pierre C. (1965). On the Nature of Allosteric Transitions: A Plausible Model. J. Mol. Biol. 12, 88–118. 14343300 10.1016/s0022-2836(65)80285-6

[B66] Jørgensen T. J. D. Roepstorff P. Heck A. J. R. (1998). Direct Determination of Solution Binding Constants for Noncovalent Complexes between Bacterial Cell Wall Peptide Analogues and Vancomycin Group Antibiotics by Electrospray Ionization Mass Spectrometry. Anal. Chem. 70 (20), 4427–4432. 10.1021/ac980563h

[B67] Juraschek R. Dülcks T. Karas M. (1999). Nanoelectrospray-More Than Just a Minimized-Flow Electrospray Ionization Source. J. Am. Soc. Mass. Spectrom. 10, 300–308. 10.1016/S1044-0305(98)00157-3 10197351

[B68] Jurneczko E. Barran P. E. (2011). How Useful Is Ion Mobility Mass Spectrometry for Structural Biology? the Relationship between Protein crystal Structures and Their Collision Cross Sections in the Gas Phase. Analyst 136 (1), 20–28. 10.1039/c0an00373e 20820495

[B69] Katta V. Chait B. T. (1991). Observation of the Heme-Globin Complex in Native Myoglobin by Electrospray-Ionization Mass Spectrometry. J. Am. Chem. Soc. 113 (13), 8534–8535. 10.1021/ja00022a058

[B70] Kaur U. Johnson D. T. Chea E. E. Deredge D. J. Espino J. A. Jones L. M. (2019). Evolution of Structural Biology through the Lens of Mass Spectrometry. Anal. Chem. 91 (1), 142–155. 10.1021/acs.analchem.8b05014 30457831 PMC6472977

[B71] Kebarle P. Peschke M. (2000). On the Mechanisms by Which the Charged Droplets Produced by Electrospray lead to Gas Phase Ions. Analytica Chim. Acta 406, 11–35. 10.1016/s0003-2670(99)00598-x

[B72] Kebarle P. Verkerk U. H. (2009). Electrospray: from Ions in Solution to Ions in the Gas Phase, what We Know Now. Mass. Spectrom. Rev. 28 (6), 898–917. 10.1002/mas.20247/full 19551695

[B73] Keener J. E. Zhang G. Marty M. T. (2021). Native Mass Spectrometry of Membrane Proteins. Anal. Chem. 93 (1), 583–597. 10.1021/acs.analchem.0c04342 33115234 PMC7855921

[B74] Khristenko N. Amato J. Livet S. Pagano B. Randazzo A. Gabelica V. (2019). Native Ion Mobility Mass Spectrometry: When Gas-phase Ion Structures Depend on the Electrospray Charging Process. J. Am. Soc. Mass. Spectrom. 30 (6), 1069–1081. 10.1007/s13361-019-02152-3 30924079

[B75] Koeniger S. L. Clemmer D. E. (2007). Resolution and Structural Transitions of Elongated States of Ubiquitin. J. Am. Soc. Mass. Spectrom. 18 (2), 322–331. 10.1016/j.jasms.2006.09.025 17084091

[B76] Konermann L. Ahadi E. Rodriguez A. D. Vahidi S. (2013). Unraveling the Mechanism of Electrospray Ionization. Anal. Chem. 85 (1), 2–9. 10.1021/ac302789c 23134552

[B77] Konermann L. Douglas D. J. (1998). Unfolding of Proteins Monitored by Electrospray Ionization Mass Spectrometry: A Comparison of Positive and Negative Ion Modes. J. Am. Soc. Mass. Spectrom. 9 (12), 1248–1254. 10.1016/S1044-0305(98)00103-2 9835071

[B78] Konermann L. Pan J. Liu Y.-H. (2011). Hydrogen Exchange Mass Spectrometry for Studying Protein Structure and Dynamics. Chem. Soc. Rev. 40 (3), 1224–1234. 10.1039/c0cs00113a 21173980

[B79] Koshland D. E. Némethy G. Filmer D. (1965). Comparison of Experimental Binding Data and Theoretical Models in Proteins Containing Subunits. Biochemistry 5 (1), 365–385. 10.1021/bi00865a047 5938952

[B80] Kyrilis F. L. Semchonok D. A. Skalidis I. Tüting C. Hamdi F. O’Reilly F. J. (2021). Integrative Structure of a 10-megadalton Eukaryotic Pyruvate Dehydrogenase Complex from Native Cell Extracts. Cel Rep. 34 (6), 108727. 10.1016/j.celrep.2021.108727 33567276

[B81] Laganowsky A. Reading E. Hopper J. T. S. Robinson C. V. (2013). Mass Spectrometry of Intact Membrane Protein Complexes. Nat. Protoc. 8 (4), 639–651. 10.1038/nprot.2013.024 23471109 PMC4058633

[B82] Lai A. L. Clerico E. M. Blackburn M. E. Patel N. A. Robinson C. V. Borbat P. P. (2017). Key Features of an Hsp70 Chaperone Allosteric Landscape Revealed by Ion-Mobility Native Mass Spectrometry and Double Electron-Electron Resonance. J. Biol. Chem. 292 (21), 8773–8785. 10.1074/jbc.M116.770404 28428246 PMC5448104

[B83] Lanucara F. Holman S. W. Gray C. J. Eyers C. E. (2014). The Power of Ion Mobility-Mass Spectrometry for Structural Characterization and the Study of Conformational Dynamics. Nat. Chem 6 (4), 281–294. 10.1038/nchem.1889 24651194

[B84] Lebrette H. Borezée-Durant E. Martin L. Richaud P. Boeri Erba E. Cavazza C. (2015). Novel Insights into Nickel Import in *Staphylococcus aureus*: the Positive Role of Free Histidine and Structural Characterization of a New Thiazolidine-type Nickel Chelator. Metallomics 7, 613–621. 10.1039/c4mt00295d 25611161

[B85] Liu L. Kitova E. N. Klassen J. S. (2011). Quantifying Protein-Fatty Acid Interactions Using Electrospray Ionization Mass Spectrometry. J. Am. Soc. Mass. Spectrom. 22 (2), 310–318. 10.1007/s13361-010-0032-5 21472590

[B86] Loo J. A. Berhane B. Kaddis C. S. Wooding K. M. Xie Y. Kaufman S. L. (2005). Electrospray Ionization Mass Spectrometry and Ion Mobility Analysis of the 20S Proteasome Complex. J. Am. Soc. Mass. Spectrom. 16 (7), 998–1008. 10.1016/j.jasms.2005.02.017 15914020

[B87] Loo R. R. O. Goodlett D. R. Smith R. D. Loo J. A. (1993). Observation of a Noncovalent Ribonuclease S-Protein/S-Peptide Complex by Electrospray Ionization Mass Spectrometry. J. Am. Chem. Soc. 115 (10), 4391–4392. 10.1021/ja00063a079

[B88] Lössl P. Snijder J. Heck A. J. R. (2014). Boundaries of Mass Resolution in Native Mass Spectrometry. J. Am. Soc. Mass. Spectrom. 25 (6), 906–917. 10.1007/s13361-014-0874-3 24700121

[B89] Lu W. Kostic M. Zhang T. Che J. Patricelli M. P. Jones L. H. (2021). Fragment-based Covalent Ligand Discovery. RSC Chem. Biol. 2 (2), 354–367. 10.1039/d0cb00222d 34458789 PMC8341086

[B90] Maple H. J. Garlish R. A. Rigau-Roca L. Porter J. Whitcombe I. Prosser C. E. (2012). Automated Protein-Ligand Interaction Screening by Mass Spectrometry. J. Med. Chem. 55 (2), 837–851. 10.1021/jm201347k 22148839

[B91] Marchand A. Czar M. F. Eggel E. N. Kaeslin J. Zenobi R. (2020). Studying Biomolecular Folding and Binding Using Temperature-Jump Mass Spectrometry. Nat. Commun. 11 (1), 1. 10.1038/s41467-019-14179-x 31992698 PMC6987177

[B92] Marchand A. Rosu F. Zenobi R. Gabelica V. (2018). Thermal Denaturation of DNA G-Quadruplexes and Their Complexes with Ligands: Thermodynamic Analysis of the Multiple States Revealed by Mass Spectrometry. J. Am. Chem. Soc. 140 (39), 12553–12565. 10.1021/jacs.8b07302 30183275

[B93] Marcoux J. Wang S. C. Politis A. Reading E. Ma J. Biggin P. C. (2013). Mass Spectrometry Reveals Synergistic Effects of Nucleotides, Lipids, and Drugs Binding to a Multidrug Resistance Efflux Pump. Proc. Natl. Acad. Sci. 110 (24), 9704–9709. 10.1073/pnas.1303888110 23690617 PMC3683783

[B94] Marklund E. G. Benesch J. L. (2019). Weighing-up Protein Dynamics: the Combination of Native Mass Spectrometry and Molecular Dynamics Simulations. Curr. Opin. Struct. Biol. 54, 50–58. 10.1016/j.sbi.2018.12.011 30743182

[B95] Marty M. T. Hoi K. K. Gault J. Robinson C. V. (2016). Probing the Lipid Annular Belt by Gas-phase Dissociation of Membrane Proteins in Nanodiscs. Angew. Chem. Int. Ed. 55 (2), 550–554. 10.1002/anie.201508289 PMC4736441 26594028

[B96] Masson G. R. Burke J. E. Ahn N. G. Anand G. S. Borchers C. Brier S. (2019). Recommendations for Performing, Interpreting and Reporting Hydrogen Deuterium Exchange Mass Spectrometry (HDX-MS) Experiments. Nat. Methods 16 (7), 595–602. 10.1038/s41592-019-0459-y 31249422 PMC6614034

[B97] McKay A. R. Ruotolo B. T. Ilag L. L. Robinson C. V. (2006). Mass Measurements of Increased Accuracy Resolve Heterogeneous Populations of Intact Ribosomes. J. Am. Chem. Soc. 128 (35), 11433–11442. 10.1021/ja061468q 16939266

[B98] Mehmood S. Allison T. M. Robinson C. V. (2015). Mass Spectrometry of Protein Complexes: From Origins to Applications. Annu. Rev. Phys. Chem. 66 (1), 453–474. 10.1146/annurev-physchem-040214-121732 25594852

[B99] Meyer B. Peters T. (2003). NMR Spectroscopy Techniques for Screening and Identifying Ligand Binding to Protein Receptors. Angew. Chem. Int. Ed. 42 (8), 864–890. 10.1002/anie.200390233 12596167

[B153] Mirgorodskaya O. A. Shevchenko A. A. Chernushevich I. V. Dodonov A. F. Miroshnikov A. I. (1994). Electrospray-Ionization Time-of-Flight Mass Spectrometry in Protein Chemistry. Analytical Chemistry 66.1, 99–107.

[B152] Morris H. R. Paxton T. Dell A. Langhorne J. Berg M. Bordoli R. S. (1996). High Sensitivity Collisionally-Activated Decomposition Tandem Mass Spectrometry on a Novel Quadrupole/Orthogonal-Acceleration Time-of-Flight Mass Spectrometer. Rapid Communications in Mass Spectrometry 10.0, 889–896. 8777321 10.1002/(SICI)1097-0231(19960610)10:8<889::AID-RCM615>3.0.CO;2-F

[B100] Nematollahi L. A. Garza-Garcia A. Bechara C. Esposito D. Morgner N. Robinson C. V. (2015). Flexible Stoichiometry and Asymmetry of the PIDDosome Core Complex by Heteronuclear NMR Spectroscopy and Mass Spectrometry. J. Mol. Biol. 427 (4), 737–752. 10.1016/j.jmb.2014.11.021 25528640 PMC4332690

[B101] Nesatyy V. J. Suter M. J.-F. (2004). On the Conformation-dependent Neutralization Theory and Charging of Individual Proteins and Their Non-covalent Complexes in the Gas Phase. J. Mass. Spectrom. 39 (1), 93–97. 10.1002/jms.522 14760619

[B102] Nguyen G. T. H. Bennett J. L. Liu S. Hancock S. E. Winter D. L. Glover D. J. (2021). Multiplexed Screening of Thousands of Natural Products for Protein-Ligand Binding in Native Mass Spectrometry. J. Am. Chem. Soc. 143, 21379–21387. 10.1021/jacs.1c10408 34886668

[B103] Niu S. Rabuck J. N. Ruotolo B. T. (2013). Ion Mobility-Mass Spectrometry of Intact Protein-Lligand Complexes for Pharmaceutical Drug Discovery and Development. Curr. Opin. Chem. Biol. 17, 809–817. 10.1016/j.cbpa.2013.06.019 23856053

[B104] Oikonomou C. M. Jensen G. J. (2017). Cellular Electron Cryotomography: Toward Structural Biology *In Situ* . Annu. Rev. Biochem. 86, 873–896. 10.1146/annurev-biochem-061516-044741 28426242

[B105] Olinares P. D. B. Kang J. Y. Llewellyn E. Chiu C. Chen J. Malone B. (2021). Native Mass Spectrometry-Based Screening for Optimal Sample Preparation in Single-Particle Cryo-EM. Structure 29 (2), 186–195. 10.1016/j.str.2020.11.001 33217329 PMC7867593

[B106] Peschke M. Verkerk U. H. Kebarle P. (2004). Features of the ESI Mechanism that Affect the Observation of Multiply Charged Noncovalent Protein Complexes and the Determination of the Association Constant by the Titration Method. J. Am. Soc. Mass. Spectrom. 15 (10), 1424–1434. 10.1016/j.jasms.2004.05.005 15465355

[B107] Politis A. Stengel F. Hall Z. Hernández H. Leitner A. Walzthoeni T. (2014). A Mass Spectrometry-Based Hybrid Method for Structural Modeling of Protein Complexes. Nat. Methods 11, 403–406. 10.1038/nmeth.2841 24509631 PMC3972104

[B108] Rathore D. Faustino A. Schiel J. Pang E. Boyne M. Rogstad S. (2018). The Role of Mass Spectrometry in the Characterization of Biologic Protein Products. Expert Rev. Proteomics 15 (5), 431–449. 10.1080/14789450.2018.1469982 29694790

[B109] Reading E. Liko I. Allison T. M. Benesch J. L. P. Laganowsky A. Robinson C. V. (2015). The Role of the Detergent Micelle in Preserving the Structure of Membrane Proteins in the Gas Phase. Angew. Chem. 127, 4660–4664. 10.1002/anie.20141162210.1002/ange.201411622 25693501

[B110] Rezaei Darestani R. Winter P. Kitova E. N. Tuszynski J. A. Klassen J. S. (2016). Screening Anti-cancer Drugs against Tubulin Using Catch-And-Release Electrospray Ionization Mass Spectrometry. J. Am. Soc. Mass. Spectrom. 27 (5), 876–885. 10.1007/s13361-016-1360-x 26944280

[B111] Robinson C. V. (2019). Mass Spectrometry: From Plasma Proteins to Mitochondrial Membranes. Proc. Natl. Acad. Sci. USA 116 (8), 2814–2820. 10.1073/pnas.1820450116 30718422 PMC6386728

[B112] Rose R. J. Damoc E. Denisov E. Makarov A. Heck A. J. R. (2012). High-sensitivity Orbitrap Mass Analysis of Intact Macromolecular Assemblies. Nat. Methods 9 (11), 1084–1086. 10.1038/nmeth.2208 23064518

[B113] Rostom A. A. Robinson C. V. (1999). Detection of the Intact GroEL Chaperonin Assembly by Mass Spectrometry. J. Am. Chem. Soc. 121 (7), 4718–4719. 10.1021/ja990238r

[B114] Rostom A. A. Fucini P. Benjamin D. R. Juenemann R. Nierhaus K. H. Hartl F. U. (2000). Detection and Selective Dissociation of Intact Ribosomes in a Mass Spectrometer. Proc. Natl. Acad. Sci. 97 (10), 5185–5190. 10.1073/pnas.97.10.5185 10805779 PMC25803

[B115] Rožman M. Gaskell S. J. (2010). Non-covalent Interactions of Alkali Metal Cations with Singly Charged Tryptic Peptides. J. Mass. Spectrom. 45 (12), 1409–1415. 10.1002/jms.1856 21031360

[B116] Ruotolo B. Robinson C. (2006). Aspects of Native Proteins Are Retained in Vacuum. Curr. Opin. Chem. Biol. 10 (5), 402–408. 10.1016/j.cbpa.2006.08.020 16935553

[B117] Ruotolo B. T. Benesch J. L. P. Sandercock A. M. Hyung S.-J. Robinson C. V. (2008). Ion Mobility-Mass Spectrometry Analysis of Large Protein Complexes. Nat. Protoc. 3 (7), 1139–1152. 10.1038/nprot.2008.78 18600219

[B118] Ruotolo B. T. Hyung S.-J. Robinson P. M. Giles K. Bateman R. H. Robinson C. V. (2007). Ion Mobility-Mass Spectrometry Reveals Long-Lived, Unfolded Intermediates in the Dissociation of Protein Complexes. Angew. Chem. Int. Ed. 46 (42), 8001–8004. 10.1002/anie.200702161 17854106

[B119] Santambrogio C. Ricagno S. Sobott F. Colombo M. Bolognesi M. Grandori R. (2011). Characterization of β2-microglobulin Conformational Intermediates Associated to Different Fibrillation Conditions. J. Mass. Spectrom. 46 (8), 734–741. 10.1002/jms.1946 21766392

[B120] Shelimov K. B. Clemmer D. E. Hudgins R. R. Jarrold M. F. (1997). Protein Structure In Vacuo: Gas-phase Conformations of BPTI and Cytochrome C. J. Am. Chem. Soc. 119 (9), 2240–2248. 10.1021/ja9619059

[B121] Slonusakiewicz J. Ng W. Dai J. Pasternak A. Redden P. (2005). Frontal Affinity Chromatography with MS Detection (FAC-MS) in Drug Discovery. Drug Discov. Today 10 (6), 409–416. 10.1016/s1359-6446(04)03360-4 15808820

[B122] Smith D. L. Deng Y. Zhang Z. (1997). Probing the Non-covalent Structure of Proteins by Amide Hydrogen Exchange and Mass Spectrometry. J. Mass. Spectrom. 32 (2), 135–146. 10.1002/(SICI)1096-9888(199702)32:2<135::AID-JMS486>3.0.CO;2-M 9102198

[B123] Smith D. P. Radford S. E. Ashcroft A. E. (2010). Elongated Oligomers in 2-microglobulin Amyloid Assembly Revealed by Ion Mobility Spectrometry-Mass Spectrometry. Proc. Natl. Acad. Sci. 107 (15), 6794–6798. 10.1073/pnas.0913046107 20351246 PMC2872402

[B124] Snijder J. Rose R. J. Veesler D. Johnson J. E. Heck A. J. R. (2013). Studying 18 MDa Virus Assemblies with Native Mass Spectrometry. Angew. Chem. Int. Ed. 52 (14), 4020–4023. 10.1002/anie.201210197 PMC3949431 23450509

[B125] Snijder J. Schuller J. M. Wiegard A. Lössl P. Schmelling N. Axmann I. M. (2017). Structures of the Cyanobacterial Circadian Oscillator Frozen in a Fully Assembled State. Science 355 (6330), 1181–1184. 10.1126/science.aag3218 28302852

[B126] Sobott F. Robinson C. V. (2006). Understanding Protein Interactions and Their Representation in the Gas Phase of the Mass Spectrometer. Princ Mass. Spectrom. Appl. Biomol. 2006, 147–175. 10.1002/047005042X.ch4

[B127] Stojko J. Fieulaine S. Petiot-Bécard S. Van Dorsselaer A. Meinnel T. Giglione C. (2015). Ion Mobility Coupled to Native Mass Spectrometry as a Relevant Tool to Investigate Extremely Small Ligand-Induced Conformational Changes. Analyst 140 (21), 7234–7245. 10.1039/C5AN01311A 26401526

[B128] Strop P. Brunger A. T. (2005). Refractive index-based Determination of Detergent Concentration and its Application to the Study of Membrane Proteins. Protein Sci. 14 (8), 2207–2211. 10.1110/ps.051543805 16046633 PMC2279333

[B129] Susa A. C. Xia Z. Williams E. R. (2017). Small Emitter Tips for Native Mass Spectrometry of Proteins and Protein Complexes from Nonvolatile Buffers that Mimic the Intracellular Environment. Anal. Chem. 89 (5), 3116–3122. 10.1021/acs.analchem.6b04897 28192954

[B130] Terral G. Beck A. Cianférani S. (2016). Insights from Native Mass Spectrometry and Ion Mobility-Mass Spectrometry for Antibody and Antibody-Based Product Characterization. J. Chromatogr. B 1032, 79–90. 10.1016/j.jchromb.2016.03.044 27108304

[B131] Thompson N. J. Rosati S. Heck A. J. R. (2014). Performing Native Mass Spectrometry Analysis on Therapeutic Antibodies. Methods 65 (1), 11–17. 10.1016/j.ymeth.2013.05.003 23688935

[B132] Thomson B. A. Iribarne J. V. (1979). Field Induced Ion Evaporation from Liquid Surfaces at Atmospheric Pressure. J. Chem. Phys. 71 (11), 4451–4463. 10.1063/1.438198

[B133] Van De Waterbeemd M. Fort K. L. Boll D. Reinhardt-Szyba M. Routh A. Makarov A. (2017). High-fidelity Mass Analysis Unveils Heterogeneity in Intact Ribosomal Particles. Nat. Methods 14 (3), 283–286. 10.1038/nmeth.4147 28114288

[B134] Vivat Hannah V. Atmanene C. Zeyer D. Van Dorsselaer A. Sanglier-Cianférani S. (2010). Native MS: an 'ESI‚ Way to Support Structure- and Fragment-Based Drug Discovery. Future Med. Chem. 2 (1), 35–50. 10.4155/fmc.09.141 21426045

[B135] Wagner S. Baars L. Ytterberg A. J. Klussmeier A. Wagner C. S. Nord O. (2007). Consequences of Membrane Protein Overexpression in *Escherichia coli* . Mol. Cell Proteomics 6 (9), 1527–1550. 10.1074/mcp.M600431-MCP200 17446557

[B136] Wilm M. Mann M. (1996). Analytical Properties of the Nanoelectrospray Ion Source. Anal. Chem. 68, 1–8. 10.1021/ac9509519 8779426

[B137] Wilm M. S. Mann M. (1994). Electrospray and Taylor-Cone Theory, Dole’s Beam of Macromolecules at Last? Int. J. Mass. Spectrom. Ion Process. 136 (2), 167–180. 10.1016/0168-1176(94)04024-9

[B138] Wolynes P. G. (1995). Biomolecular Folding In Vacuo!!!(?). Proc. Natl. Acad. Sci. 92 (7), 2426–2427. 10.1073/pnas.92.7.2426 7708658 PMC42230

[B139] Woods L. A. Dolezal O. Ren B. Ryan J. H. Peat T. S. Poulsen S. A. (2016). Native State Mass Spectrometry, Surface Plasmon Resonance, and X-ray Crystallography Correlate Strongly as a Fragment Screening Combination. J. Med. Chem. 59, 2192–2204. 10.1021/acs.jmedchem.5b01940 26882437

[B140] Wortmann A. Jecklin M. C. Touboul D. Badertscher M. Zenobi R. (2008). Binding Constant Determination of High-Affinity Protein-Ligand Complexes by Electrospray Ionization Mass Spectrometry and Ligand Competition. J. Mass. Spectrom. 43 (5), 600–608. 10.1002/jms.1355 18074334

[B141] Wu C. Klasmeier J. Hill H. H. (1999). Atmospheric Pressure Ion Mobility Spectrometry of Protonated and Sodiated Peptides. Rapid Commun. Mass. Spectrom. 13 (12), 1138–1142. 10.1002/(sici)1097-0231(19990630)13:12<1138::aid-rcm625>3.0.co;2-8 10390859

[B142] Wyttenbach T. Bowers M. T. (2011). Structural Stability from Solution to the Gas Phase: Native Solution Structure of Ubiquitin Survives Analysis in a Solvent-free Ion Mobility-Mass Spectrometry Environment. J. Phys. Chem. B 115 (42), 12266–12275. 10.1021/jp206867a 21905704

[B143] Yee A. W. Aldeghi M. Blakeley M. P. Ostermann A. Mas P. J. Moulin M. (2019). A Molecular Mechanism for Transthyretin Amyloidogenesis. Nat. Commun. 10 (1), 1–10. 10.1038/s41467-019-08609-z 30804345 PMC6390107

[B144] Yen H.-Y. Hoi K. K. Liko I. Hedger G. Horrell M. R. Song W. (2018). PtdIns(4,5)P2 Stabilizes Active States of GPCRs and Enhances Selectivity of G-Protein Coupling. Nature 559, 423–427. 10.1038/s41586-018-0325-6 29995853 PMC6059376

[B145] Yen H. Y. Hopper J. T. S. Liko I. Allison T. M. Zhu Y. Wang D. (2017). Ligand Binding to a G Protein-Coupled Receptor Captured in a Mass Spectrometer. Sci. Adv. 3 (6), e1701016–6. 10.1126/sciadv.1701016 28630934 PMC5473672

[B146] Young L. M. Saunders J. C. Mahood R. A. Revill C. H. Foster R. J. Tu L.-H. (2014). Screening and Classifying Small-Molecule Inhibitors of Amyloid Formation Using Ion Mobility Spectrometry-Mass Spectrometry. Nat. Chem 7 (1), 73–81. 10.1038/nchem.2129 25515893 PMC4280571

[B147] Zhang H. Cui W. Wen J. Blankenship R. E. Gross M. L. (2011). Native Electrospray and Electron-Capture Dissociation FTICR Mass Spectrometry for Top-Down Studies of Protein Assemblies. Anal. Chem. 83 (14), 5598–5606. 10.1021/ac200695d 21612283 PMC3136621

[B148] Zhang S. Van Pelt C. K. Wilson D. B. (2003). Quantitative Determination of Noncovalent Binding Interactions Using Automated Nanoelectrospray Mass Spectrometry. Anal. Chem. 75 (13), 3010–3018. 10.1021/ac034089d 12964745

[B151] Zhong Y. Hyung S. J. Ruotolo B. T. (2012). Ion Mobility-Mass Spectrometry for Structural Proteomics. Expert Rev. Proteomics 9, 47–58. 22292823 10.1586/epr.11.75 PMC3313071

[B149] Zhou M. Sandercock A. M. Fraser C. S. Ridlova G. Stephens E. Schenauer M. R. (2008). Mass Spectrometry Reveals Modularity and a Complete Subunit Interaction Map of the Eukaryotic Translation Factor eIF3. Proc. Natl. Acad. Sci. 105 (47), 18139–18144. 10.1073/pnas.0801313105 18599441 PMC2587604

[B150] Zweers J. C. Wiegert T. van Dijl J. M. (2009). Stress-responsive Systems Set Specific Limits to the Overproduction of Membrane Proteins in Bacillus Subtilis. Appl. Environ. Microbiol. 75 (23), 7356–7364. 10.1128/AEM.01560-09 19820159 PMC2786430

